# Distinct soluble immune checkpoint profiles characterize COVID-19 severity, mortality and SARS-CoV-2 variant infections

**DOI:** 10.3389/fimmu.2024.1464480

**Published:** 2024-09-23

**Authors:** Tudorita Gabriela Paranga, Mariana Pavel-Tanasa, Daniela Constantinescu, Elena Iftimi, Claudia Elena Plesca, Ionela-Larisa Miftode, Petru Cianga, Egidia Miftode

**Affiliations:** ^1^ Department of Infectious Diseases (Internal Medicine II), Faculty of Medicine, Grigore T. Popa University of Medicine and Pharmacy, Iasi, Romania; ^2^ St. Parascheva Clinical Hospital for Infectious Diseases, Iasi, Romania; ^3^ Department of Immunology, Faculty of Medicine, Grigore T. Popa University of Medicine and Pharmacy, Iasi, Romania; ^4^ Laboratory of Immunology, St. Spiridon County Clinical Emergency Hospital, Iasi, Romania

**Keywords:** COVID-19, soluble immune checkpoints, sCD40, STIM-1, galectin-9, neural network, mortality

## Abstract

**Introduction:**

Over the past four years, the COVID-19 pandemic has posed serious global health challenges. The severe form of disease and death resulted from the failure of immune regulatory mechanisms, closely highlighted by the dual proinflammatory cytokine and soluble immune checkpoint (sICP) storm. Identifying the individual factors impacting on disease severity, evolution and outcome, as well as any additional interconnections, have become of high scientific interest.

**Methods:**

In this study, we evaluated a novel panel composed of ten sICPs for the predictive values of COVID-19 disease severity, mortality and Delta vs. Omicron variant infections in relation to hyperinflammatory biomarkers. The serum levels of sICPs from confirmed SARS-CoV-2 infected patients at hospital admission were determined by Luminex, and artificial neural network analysis was applied for defining the distinct patterns of molecular associations with each form of disease: mild, moderate, and severe.

**Results:**

Notably, distinct sICP profiles characterized various stages of disease and Delta infections: while sCD40 played a central role in all defined diagrams, the differences emerged from the distribution levels of four molecules recently found and relatively less investigated (sCD30, s4-1BB, sTIM-1, sB7-H3), and their associations with various hematological and biochemical inflammatory biomarkers. The artificial neural network analysis revealed the prominent role of serum sTIM-1 and Galectin-9 levels at hospital admission in discriminating between survivors and non-survivors, as well as the role of specific anti-interleukin therapy (Tocilizumab, Anakinra) in improving survival for patients with initially high sTIM-1 levels. Furthermore, strong associations between sCD40 and Galectin-9 with suPAR defined the Omicron variant infections, while the positive match of sCD40 with sTREM-1 serum levels characterized the Delta-infected patients.

**Conclusions:**

Of importance, this study provides a comprehensive analysis of circulatory immune factors governing the COVID-19 pathology, and identifies key roles of sCD40, sTIM-1, and Galectin-9 in predicting mortality.

## 1 Introduction

Even though four years have passed since the outbreak of the COVID-19 pandemic, it continues to pose a global health challenge. The clinical features of SARS-CoV-2 infected patient are well documented, ranging from asymptomatic, mild, and moderate to critically ill patients who present acute respiratory distress syndrome (ARDS), septic shock, metabolic acidosis, coagulopathy, organ failure, and eventually death (1–3). The fate of the COVID-19 disease is decided by T cells, which have been an important component of the cell-mediated immune responses (4–7). Despite a general reduction in the lymphocyte count, the CD4+ and CD8+ T lymphocyte subsets are hyperactivated (1). In mild COVID-19, SARS-CoV-2 induces a memory and Th1 phenotype with CD4+ T cells expressing higher levels of IFN-γ, IL-2 and TNF and a strong response of CD8+ T cells with high expression of granzyme A, B, and perforins (8). By contrast, in severe COVID-19 patients, the immune responses are marked by exhausted T cells displaying high levels of terminal differentiation/senescence markers (immune checkpoints like Programmed Death-1 (PD-1), T cell immunoglobulin and mucin domain-containing protein 3 [TIM-3), T cell immunoreceptor with Ig and ITIM domains (TIGIT)], with proinflammatory CD25+ CD8+ T cells of reduced cytotoxic potential dominating the T cell profile (9, 10). The immunological profile in ARDS is further exacerbated by the cytokine storm, which mainly arises from deficiencies in immune regulatory mechanisms. Regulatory T cells (Tregs) are central in maintaining the immune homeostasis due to their immunosuppressive potential of inhibiting excessive immune responses. Interestingly, despite a significant expansion of Tregs in severe cases compared to mild ones, their functionality is deranged. They exhibit similarities with tumor-infiltrating Tregs, characterized by a distinct transcriptional signature marked by upregulation of FoxP3, overexpression of both suppressive effectors (IL-10) and proinflammatory molecules (IL-32), and apoptotic features (11–13). As a feedback loop, components of the proinflammatory storm (IL-6 and IL-18) contribute to and potentiate this disturbed Treg phenotype (12).

The up-listed dysfunctions in immune cells characterizing the severe COVID-19 patients are associated with alterations in immune checkpoints (ICPs). ICPs comprise paired receptor-ligand molecules with co-stimulatory, inhibitory or dual effects on immune activity, defense, regulation or self-tolerance (14, 15). The up-regulation of inhibitory ICP expression on various immune cells, such as T and B lymphocytes and NK cells, and their increased interaction with specific membrane or soluble ligands in tissue endothelium, antigen-presenting cells (APCs), and plasma are tightly linked to their exhaustion and depletion in a variety of viral infections, including COVID-19 (16, 17). Of note, soluble isoforms of immune checkpoints (sICPs) may result from the protease-mediated shedding of the membrane ICPs(mICPs) or by alternative mRNA splicing, and likely form a complex circulating immune regulatory system (18, 19). On one hand, the blood levels of sICPs may reflect the extent of expression of their membrane-bound counterparts, but interacting with their specific membrane receptors or ligands, these ICPs may potentiate or inhibit the activity of various immune cells. Indeed, a storm of sICPs was associated with severe COVID-19 and their levels on hospital admission were suggested they might serve as biomarkers of disease progression and outcome (19, 20). Thus, understanding the profile of soluble immune checkpoints in COVID-19 patients with distinct forms of disease severity, ranging from mild to moderate and severe, and their potential interlinks with multiple inflammatory mediators would provide a broad picture of the complex immune processes underlying the disease pathogenesis, and potentially predict the individual pattern of evolution and outcome. These observations would be crucial in the management of the SARS-CoV-2 infected patients and for applying the most effective therapeutic strategy.

Based on these considerations, we set up the evaluation of a novel sICPs panel composed of commonly investigated sICPs (including sCD40 with its ligands sCD40L and Gal-9; sCD25; sCD27) as well as molecules recently found and relatively underexplored (such as sCD30, s4-1BB, sTIM-1, sB7-H3 and sCD163), in relation to previously investigated inflammatory mediators (3) and clinical characteristics of SARS-CoV-2-infected patients (for instance, the vaccination status, the presence of comorbidities, and the administered therapy during hospitalization). Here we have identified distinct baseline sICPs profiles at hospital admission characterizing the 3 forms of disease severity (mild, moderate and severe), the Delta *vs.* Omicron variant infections, and the non-survivors’ group of COVID-19 patients. While sCD40 is central in all defined profiles, the variations in distribution of four sICPs (sCD30, s4-1BB, sTIM-1, sB7-H3) and their interactions with additional paraclinical and clinical variables help distinguishing between the different subgroups of COVID-19 patients. Applying an artificial neural network analysis to our complex patients’ data, retrospectively revealed the importance of administering targeted anti-inflammatory therapy (Tocilizumab or Anakinra) in defining the outcome of patients with high baseline of sTIM-1 levels.

## 2 Methods

### 2.1 Patients’ selection and general characteristics

This study involved a retrospective analysis of prospectively collected blood samples from 153 individuals confirmed with SARS-CoV-2 infection by qRT-PCR tests through nasopharyngeal and oropharyngeal swab samples and admitted at “St. Parascheva” Clinical Hospital for Infectious Diseases (Iasi, Romania) between October 2021 and May 2022. The study included adult patients who needed hospitalization of either gender and irrespective of their vaccination status (yes/no). The patients with anti-inflammatory medication administered prior to hospital admission, immunocompromised (cancer, HIV/AIDS, transplant), pregnant or with autoimmune diseases were excluded from the study, as detailed in our previous manuscript (3). Additionally, since the first two cases of Omicron infection were officially reported in Romania on the 4^th^ of December 2021, all the cases before 1^st^ of December were designated as Delta variant infections. Since more than 60% of the nasopharyngeal/oropharyngeal swab samples sequenced by RNIPH were reported as Omicron variant starting with 1^st^ of January 2022, all the cases after this date were categorized as predominantly Omicron infections. Based on these considerations, the cases from December 2021 were thus excluded from our analysis.

The patients were stratified according to disease severity in mild, moderate and severe cases according to the international clinical spectrum guidelines of SARS-CoV-2 infection, detailed in ratio (3). The general patients’ characteristics are detailed in **Supplementary Table 1**. Aliquots from the blood samples were previously analyzed for investigating the inflammatory profile of the COVID-19 patients stratified according to disease severity or SARS-CoV-2 variant infections: CRP (C-reactive protein), suPAR (soluble urokinase plasminogen activator receptor), sTREM-1 (soluble form of triggering receptor expressed on myeloid cells 1), MCP-1/CCL2 (monocyte chemoattractant protein-1), HGF (hepatocyte growth factor), IL-1β, IL-6, NLR (neutrophil-lymphocyte ratio), PLR (platelet-lymphocyte ratio), fibrinogen, ferritin, LDH (lactate dehydrogenase) (3).

Participation in the study did not affect patient management, and attending physicians retained full discretion over therapeutic decisions.

The study was reviewed and approved by Institutional Ethics Committee – Grigore T. Popa University of Medicine and Pharmacy of Iasi, no. 119/31.10.2021. The patients/participants provided their written informed consent to participate in this study.

### 2.2 Quantification of soluble immune checkpoints

The blood samples were collected within 4 hours of hospital admission in vacutainers with no anticoagulant and processed within 6 hours of receipt. The blood was spun at 2000 G for 5 min, and the serum was separated in multiple aliquots of 500 μl which were stored at -80°C until further analysis, as detailed in (3).

The serum concentration of distinct immune checkpoint molecules was performed using a human custom pre-mixed multi-analyte kit from R&D systems and performed on a Luminex 100/200 analyzer in the Laboratory of Immunology of the “St. Spiridon” Emergency County Hospital, Iasi. A fresh aliquot of each patient sample, previously stored at -80^0^C, was thawed and centrifuged at 2,000 G for 5 min. The supernatants were 2-fold diluted using the calibrator diluent RD6-52 prior to processing as per the manufacturer’s guidelines. The investigated biomarkers included the soluble forms of: CD40/TNFRSF5, CD27/TNFRSF7, CD30/TNFRSF8, 4-1BB/TNFRSF9/CD137, CD25/IL-2Rα, TIM-1/KIM-1/HAVCR, CD40 Ligand/TNFSF5, Galectin-9, B7-H3/CD276, and CD163/M130/p155. Importantly, the kit allowed the identification of total Galectin-9 levels, including both full-length and truncated isoforms. Briefly, 50 μl of magnetic microparticle cocktail were incubated with 50 μl of standards and prediluted samples in designated 96-well plates for 2 hours at room temperature on a horizontal orbital microplate shaker at 500 rpm. Following the washing procedure (which included 3 separate washes and the use of a magnetic device designed to accommodate 96-well plates), the microparticles were further incubated with 50 μl of diluted biotin-antibody solution for 1 hour at room temperature on a shaker at 500 rpm. After another washing procedure, 50 μl of the diluted streptavidin-PE solution were added for an incubation step of 30 min. The microparticles were next resuspended in 100 μl of washing buffer and the plates were read within 60 minutes.

The quantification of serum concentrations of various inflammatory cytokines/chemokines (suPAR, sTREM-1, MCP-1/CCL-2, HGF, IL-1β, IL-6) was performed in our previous study (3). The values of additional hematological and biochemical markers were collected retrospectively from patients’ electronic charts, while the clinical variables were prospectively investigated by clinical consult and anamnesis.

### 2.3 Statistical analysis

Statistical analysis was performed using SPSS, v25 (IBM SPSS Software, Chicago, IL, USA) and Graph Pad Prism, v5 (Graph Pad Software, San Diego, CA, USA). Data are presented as scatter dot graphs, or box and whiskers plots. Each figure legend contains the relevant statistical information: the n, total number of participants, the significance *p*-value with the applied statistical tests. All data were checked for both normality and variance using the Shapiro-Wilk test prior further analysis with parametric tests (unpaired t-test and one-way ANOVA with *Post-hoc* Tukey’s Multiple Comparison test) or non-parametric statistical tests, such as Mann-Whitney test (the non-parametric counterpart to unpaired t-test), and Kruskal-Wallis with Dunn’s Multiple Comparison test (the non-parametric counterpart to one-way ANOVA). The positive or negative associations between measured variables were assessed using the Spearman’s correlation coefficients (R). R values between 0.2-0.39 were treated as weak, between 0.4-0.59 as moderate, between 0.6-0.79 as very strong and above 0.8 as excellent correlation factors. The coefficient of determination R-squared (R2) was used as a goodness-of-fit measure and the F-test to determine the level of significance for each linear regression. The receiver operating characteristic (ROC) curves were generated in SPSS Statistics, v25 and the area under those curves (AUC) was used as a measure of test performance. The optimal cut-off values were determined as previously described in (21). The Kaplan-Meier survival curves, together with the univariate and multivariate analysis, and neural network models were also performed in SPSS, v25. A modern feedforward artificial neural network (multilayer perceptron/MLP) was used to investigate and reveal the existence of non-linear relationships interconnecting our patients’ clinical and paraclinical variables. To enable the comparison between multiple generated models and minimize overfitting, we performed a random assignment of 105 cases for training (68.6%) and 48 samples (31.4%) for testing prior MLP implementation. The hidden layer included 3 units and a Sigmoid activation function, while the output layer included 2 units and Softmax as activation function. Random forest models, detailed in (22), were created in Spyder 5.0 (Python 13.2) utilizing the RandomForestClassifier meta-estimator (n_estimators = 8) from the scikit-learn module. The decision tree models were generated using SPSS Statistics, v25, based on the Classification and Regression Tree (CRT) growing method, where a minimum number of 8 cases for the parent node, and 4 cases for the child node were established. The *p*-values less than 0.05 were considered statistically significant.

## 3 Results

### 3.1 COVID-19 cohort description

This study involved a retrospective analysis of prospectively collected blood samples from 153 confirmed SARS-CoV-2 infected patients upon hospital admission from October 2021 to May 2022. The blood samples have previously been investigated for the presence of a hyperinflammatory phenotype and any other disparities linked to disease severity and mortality or SARS-CoV-2 variant infection (Delta *vs.* Omicron) (3). The majority of COVID-19 cases fell into the moderate (71 patients [46.4%]) and severe (68 of patients [44.4%]) categories, with only 9.2% mild cases (14 patients). Importantly, 98 patients in our cohort were Delta variant-infected and the rest of 55 cases were categorized as Omicron infections. If the severe cases accounted for only 34.5% among Omicron infections, then, an even distribution of severe (50% of cases) and non-severe (the remaining 50% comprised by mild and moderate cases) characterized the Delta variant cohort. All mild cases survived during hospitalization, while the death rate among moderate cases was 2.8% and considerably higher among severe cases of 26.5% (*p* = 0.0012, **Supplementary Table 1**) with significant differences between Delta and Omicron infections (*p* = 0.0012). Additionally, no notable differences in age distribution (*p* = 0.1786) and female-to-male ratio (*p* = 0.4071) were observed between our groups of investigated COVID-19 patients.

### 3.2 Soluble forms of immune checkpoint molecules CD40, CD25 and Galectin-9 associate with the severe form of COVID-19

**Figure 1:**
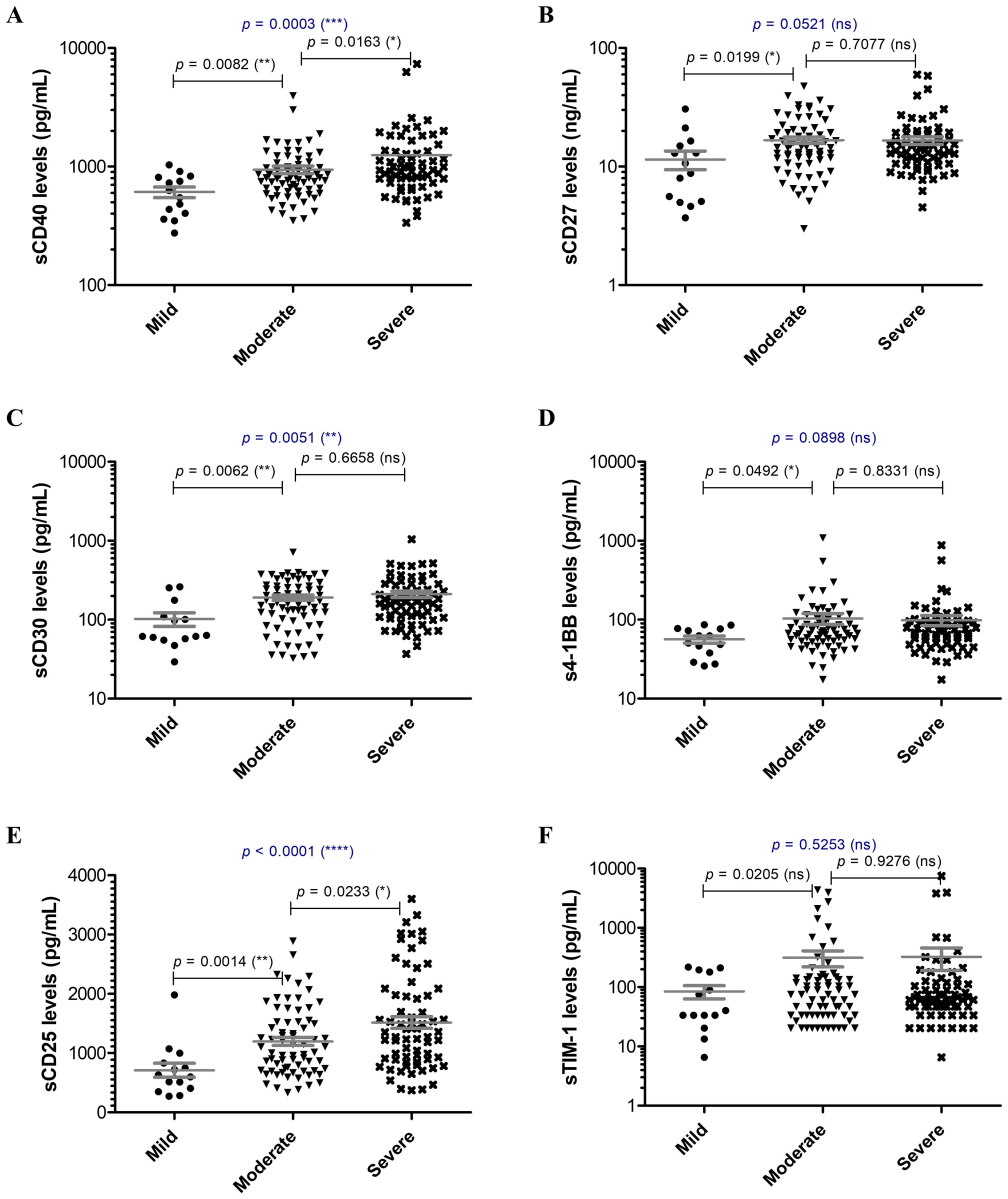
Serum profile of soluble immune checkpoint receptors in mild, moderate and severe COVID-19 disease. Serum levels of **(A)** sCD40, **(B)** sCD27, **(C)** sCD30, **(D)** s4-1BB, **(E)** sCD25, **(F)** sTIM-1 for each category of COVID-19 disease: mild, moderate or severe. The gray lines represent the mean ± SEM (****p < 0.0001, ***p < 0.001, **p < 0.01, *p < 0.05, ns – not significant; Kruskal-Wallis with Dunn’s Multiple Comparison test).

**Figure 2:**
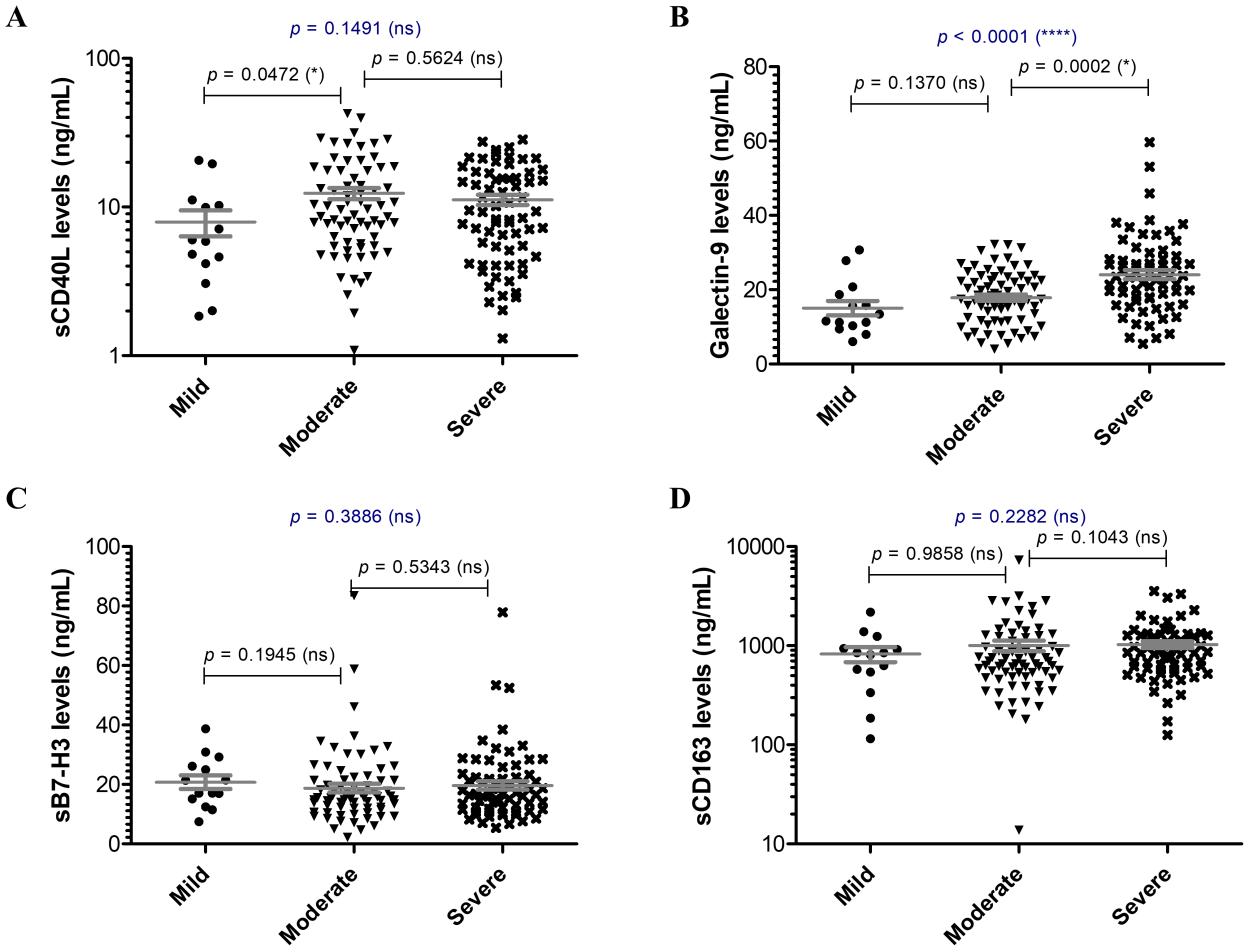
Serum profile of soluble immune checkpoint ligands and sCD163 in mild, moderate and severe COVID-19 disease. Serum levels of **(A)** sCD40L, **(B)** Galectin 9, **(C)** sB7-H3, **(D)** sCD163 for each category of COVID-19 disease: mild, moderate or severe. The gray lines represent the mean ± SEM (****p < 0.0001, *p < 0.05, ns – not significant; Kruskal-Wallis with Dunn’s Multiple Comparison test).

Since the hyperinflammatory phenotype and the different Th responses were intensively investigated for COVID-19, we aimed to extend our analysis towards the study of soluble immune checkpoint molecules (sICPs) which are believed to bridge between T and B cell activation, and the initiation of hyperinflammation. From this perspective, both soluble immune checkpoint receptors (sICRs) and ligands (sICLs) were included in our study along with the marker of macrophage activation, the soluble receptor CD163 (23). Among the investigated soluble forms of co-stimulatory ICRs belonging to the tumor necrosis factor receptor (TNFR) family (including sCD40, sCD27, sCD30, s4-1BB, **Figures 1A–D**), only the sCD40 serum levels showed a strong relation to disease severity, progressively increasing from mild to severe cases with median values of 586.3 pg/mL, 803.2 pg/mL, and 934.2 pg/mL for the three distinct groups of disease severity (*p* = 0.0003, **Figure 1A**). Importantly, sCD25, the soluble form of IL2Rα expressed on activated T cells and regulatory T cells (Tregs) followed a similar rising pattern towards severe cases (*p* < 0.0001, **Figure 1E**). Additionally, sTIM-1 (soluble T-cell immunoglobulin and mucin protein-1), the soluble form of the co-stimulatory T cell receptor TIM-1 (TIM-1) known to be a key regulator of Th2 responses, showed at least 3-fold increase in moderate and severe COVID-19 cases (**Figure 1F**). Interestingly, among sICLs, the soluble variants of both co-stimulatory molecules CD40L and Galectin-9 exhibited elevated levels in severe COVID-19 patients (**Figures 2A, B**), whereas the soluble form of the co-inhibitory molecule B7-H3 did not display any noticeable pattern (*p* = 0.3886, **Figure 2C**), and, if any, a reduction in its expression in patients with severe COVID-19. While sCD40L showed similar increased levels among severe and moderate cases, Galectin-9 was specifically elevated in the serum of severe COVID-19 subjects (*p* < 0.0001, **Figure 2B**). Interestingly, sCD163 did not display any noticeable difference among the three distinct groups of disease severity (*p* = 0.2282, **Figure 2D**).

Next, we aimed to identify whether any of these soluble immune checkpoint molecules may predict the disease severity. The ROC analysis revealed that only Galectin-9 serum levels may yield an AUC value over 0.700, with sCD40 and sCD25 giving significant but moderate predictive values for severe COVID-19 (**Supplementary Table 2**). The analysis of routinely investigated laboratory biomarkers and clinical characteristics confirmed that proinflammatory markers (CRP, ESR, ferritin, LDH), coagulation and fibrinolysis markers (e.g., fibrinogen, D-dimer), immune cells (neutrophils, eosinophils, lymphocytes, monocytes) and Delta variant infection along with age may predict disease severity (**Supplementary Table 3**). Incorporating these parameters in the model of soluble ICP molecules notably enhanced the AUC value (0.856, *p* < 0.001), and the inclusion of all patient’s characteristic only resulted in a marginal improvement of predicting disease severity (**Supplementary Figure 1**). Importantly, no parameter emerged as an independent predictive factor for disease severity in the multivariate analysis (**Supplementary Table 4**).

### 3.3 Soluble immune checkpoint ligands sCD40L, Galectin-9 and sB7-H3 distinguish between Delta and Omicron variant infections

**Figure 3:**
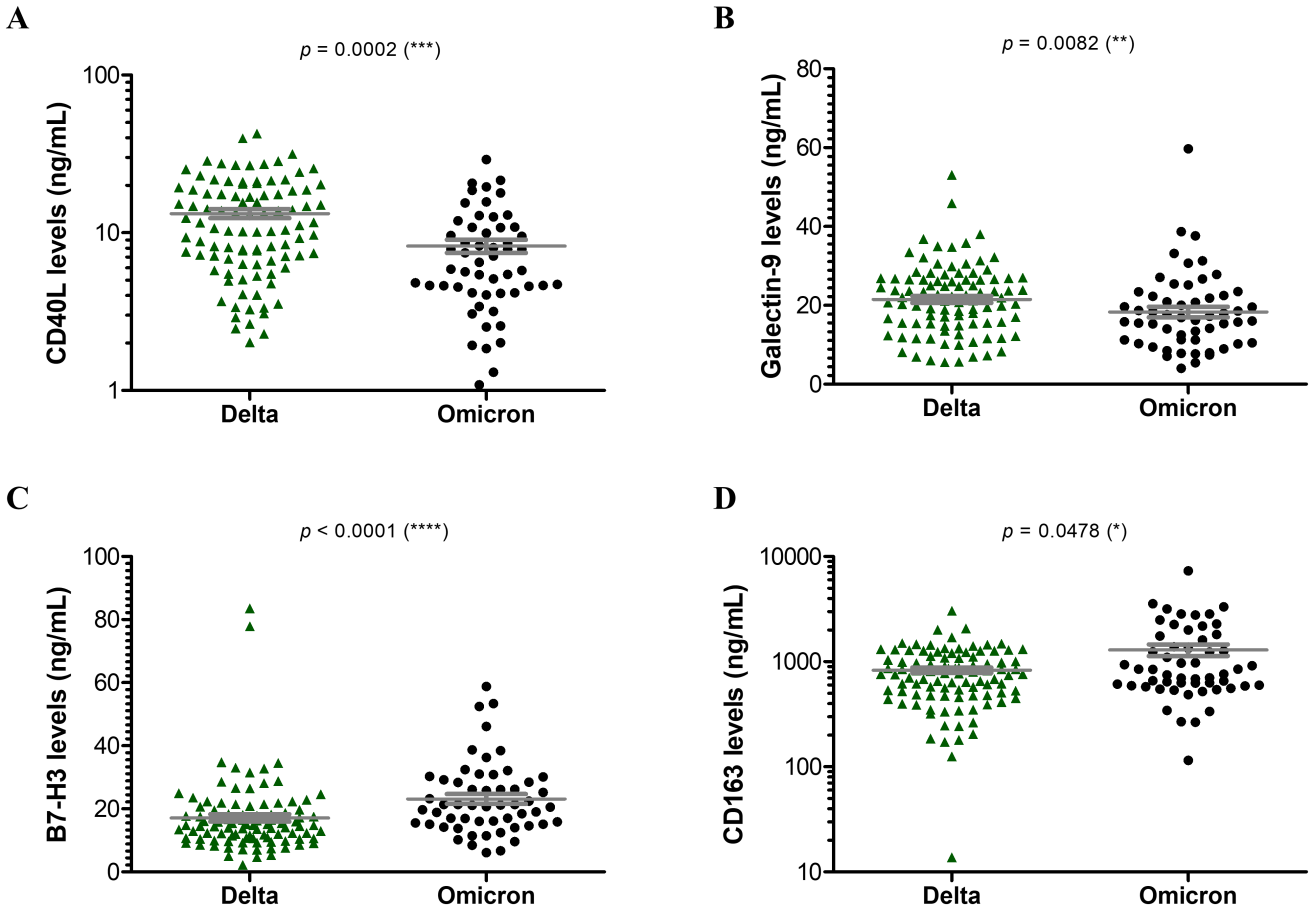
Serum profile of soluble immune checkpoint ligands and sCD163 in Delta and Omicron SARS-CoV-2 infections. Serum levels of **(A)** sCD40L, **(B)** Galectin 9, **(C)** sB7-H3, **(D)** sCD163 for each category of SARS-CoV-2 infection: Delta or Omicron. The gray lines represent the mean ± SEM (****p < 0.0001, ***p < 0.001, **p < 0.01, *p < 0.05; Kruskal-Wallis with Dunn’s Multiple Comparison test).

**Figure 4:**
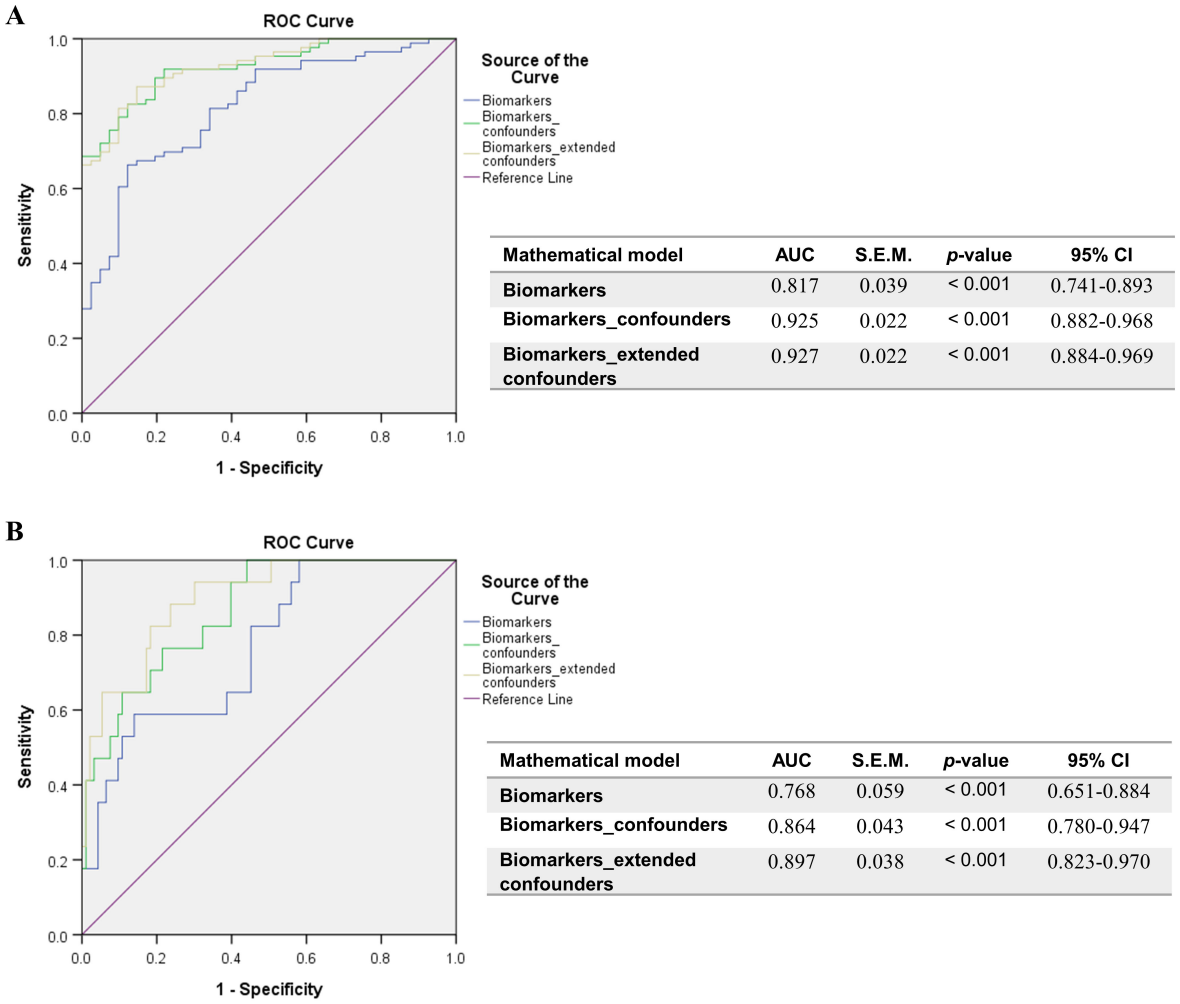
ROC curves resulted from the models comprising the positive determinants of Delta infection or COVID-19 mortality, and corrected for confounders. **(A)** Biomarkers of Delta infection: sCD30, sTIM-1, sCD40L, Galectin-9, sB7-H3, sCD163. General confounders are described in **Supplementary Table 7**, while extended confounders additionally include all patient characteristics described in **Supplementary Table 6**. **(B)** Biomarkers of COVID-19 mortality: sCD40, sCD30, sCD25, Galectin-9. General confounders are described in **Supplementary Table 10**, while extended confounders additionally include all patient characteristics described in **Supplementary Table 9**. The AUC values between 0.7-0.8 define a very good discrimination, the AUC values > 0.8 denote an excellent discrimination and the AUC values > 0.9 denote an outstanding capacity of prediction.

Further, when comparing the Delta and Omicron infections, no significant differences emerged among soluble ICRs (**Supplementary Figure 2**). By contrast, the soluble ICLs showed marked changes: the Delta infections were characterized by at least 25% increase in sCD40L (**Figure 3A**) and Gal-9 (**Figure 3B**) and 50% reduction in sB7-H3 levels (**Figure 3C**) compared to Omicron-infected subjects. Interestingly, sCD163 showed a similar trend as sB7-H3 (**Figure 3D**). In both Delta and Omicron variant infections, sB7-H3 showed a moderate correlation with sCD163 (R = 0.30, *p* = 0.0027 for Delta; R = 0.41, *p* = 0.0018 for Omicron), while only in Omicron-infected subjects Gal-9 displayed a positive association with both sB7-H3 (R = 0.54, *p* < 0.0001) and sCD163 (R = 0.37, *p* = 0.0056). Some of these observations were also supported by the ROC analysis, which identified moderate positive predictors like sCD30, sTIM-1, sCD40L, Gal-9 and negative predictors such as sB7-H3 and sCD163 for Delta infections (**Supplementary Table 5**). Considered together, these soluble ICP molecules yielded an improved AUC of 0.817 (*p* < 0.001), and, adding into the models the general parameters which also gave significant prediction values (**Supplementary Table 6**), led to an excellent AUC prediction value of 0.925 (*p* < 0.001) (**Figure 4A**). When evaluating these parameters in a multivariate analysis, several independent positive (Gal-9, potassium levels, no vaccination anti-SARS-CoV-2) and negative (sCD163, anemia) predictors for Delta infection emerged (**Supplementary Table 7**).

### 3.4 Artificial neural network models identify sCD40, Galectin-9, sTIM-1 and sCD30 as key determinants of mortality prediction in COVID-19

**Figure 5:**
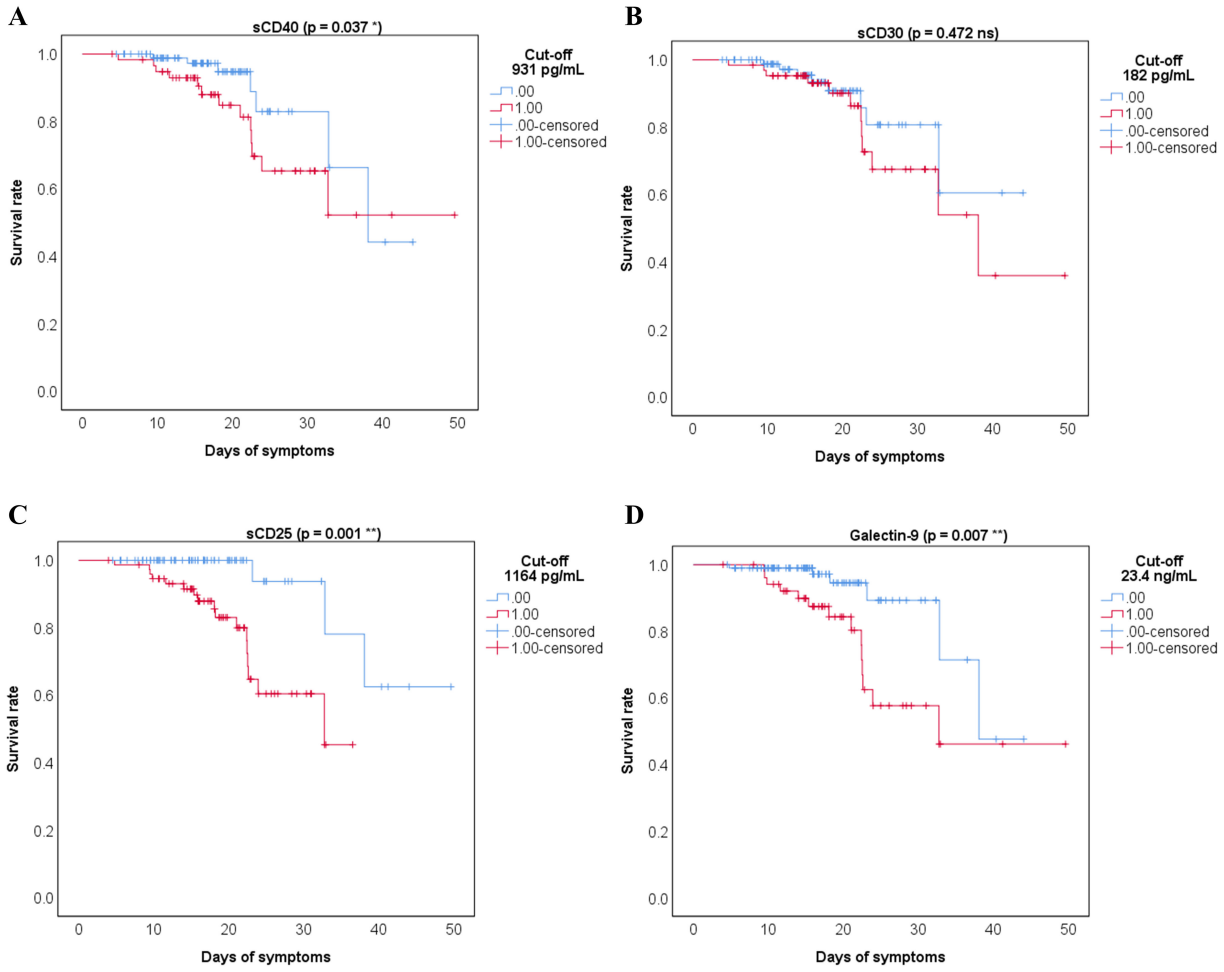
Kaplan Meier survival curves for the indicated cut-off values of the soluble immune checkpoints receptors and ligands: **(A)** sCD40, **(B)** sCD30, **(C)** sCD25, **(D)** Galectin-9. 0 = bellow cut-off value, 1 = above cut-off value (**p < 0.01, *p < 0.05, ns – not significant; Wald test).

When examining the impact on mortality by ROC regression analysis, several soluble ICP molecules proved moderate predictive capacity (sCD40, sCD30, sCD25) with Gal-9 providing the highest AUC value of 0.773 (*p* < 0.001, **Supplementary Table 8**). Since the highest death rate was observed for the severe group of COVID-19 patients (**Supplementary Table 1**), we next compared the initial serum levels of these soluble ICPs between survivors and non-survivors within this group. Importantly, sCD40, sCD30, sCD27, and Gal-9 showed a significant 30%-70% increase in the non-survivors’ cohort (**Supplementary Figures 3**, **4**). Interestingly the model arising from these variables yielded only a moderate improvement in our prediction value, and only when correcting for confounders (**Supplementary Table 9**), the AUC reached values above 0.850 (**Figure 4B**). Among all analyzed variables, LDH proved to be an independent predictor for disease mortality (**Supplementary Table 10**). While correcting for disease duration, the univariate Cox regression analysis validated all the previous findings, and the multivariate Cox analysis identified Gal-9 as a positive independent predictor (**Table 1**). Among all predictors, the identified cut-off values for sCD25 (1164 pg/mL) and Gal-9 (23.4 ng/mL) best discriminated between survivors and non-survivors, as revealed by the Kaplan-Meier curves (**Figure 5**).

**Table 1:** Univariate and multivariate Cox regression analysis of soluble IC molecules in COVID-19 patients.

Variable	Univariate analysis			Multivariate analysis		
	HR	95% CI	*p*-value	HR	95% CI	*p*-value
**sCD40 (pg/mL)**	1.000	1.000-1.001	**0.001**	1.000	0.999-1.001	0.896
**sCD27 (ng/mL)**	1.051	1.015-1.090	**0.006**	0.987	0.907-1.075	0.771
**sCD30 (pg/mL)**	1.002	1.000-1.004	**0.018**	1.002	0.999-1.005	0.151
s4-1BB (pg/mL)	1.002	1.000-1.005	0.098			
**sCD25 (pg/mL)**	1.001	1.000-1.001	**0.023**	1.000	0.999-1.001	0.630
sTIM-1 (pg/mL)	0.999	0.996-1.002	0.509			
sCD40L (ng/mL)	0.983	0.929-1.040	0.544			
**Galectin-9 (ng/mL)**	1.075	1.037-1.114	**< 0.001**	1.085	1.012-1.162	**0.021**
sB7-H3 (ng/mL)	1.013	0.987-1.040	0.336			
sCD163 (ng/mL)	1.000	1.000-1.001	0.717			

sTIM-1, soluble T cell immunoglobulin domain and the mucin domain protein-1, sCD40L, soluble CD40 ligand; HR, hazard ratio; CI, confidence interval; *p*, statistical significance coefficient. Significant p-values are highlighted in bold.

**Figure 6:**
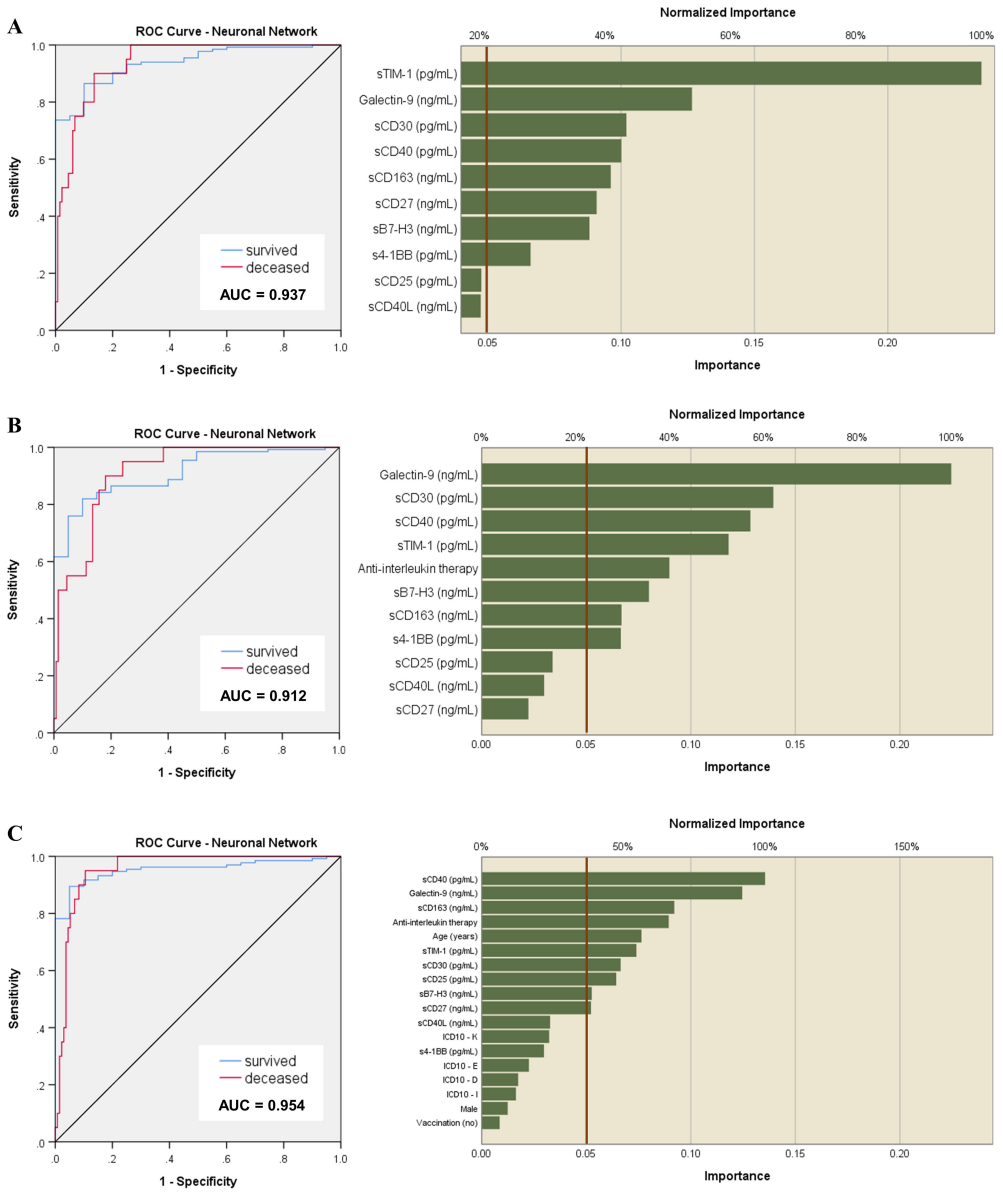
Neural network models of outcome prediction. **(A)** ROC analysis of the neural network model based on the serum levels of soluble immune checkpoint molecules and their normalized importance. Accuracy of prediction was 93.8% compared to 90.5% in the training set. **(B)** ROC analysis of the neural network model based on the serum levels of soluble immune checkpoint molecules corrected for the therapy administered during hospitalization. Accuracy of prediction was 89.6% compared to 87.6% in the training set. **(C)** ROC analysis of the previous neural network models corrected for the therapy received during hospitalization, the presence of comorbidities, vaccination status, age and gender. Accuracy of prediction was 89.6% compared to 86.7% in the training set.

Since the linear classical statistical models provided less accurate prediction capacity for mortality than for disease severity or Delta variant infection, we next aimed to use artificial neural networks (ANN) to discern complex patterns within our data and to harness the predictive potential of our biomarkers to anticipate patient outcomes. Unlike linear models, which assume a linear relationship between predictors and outcomes, the non-linear models of neural network analysis can capture intricate and non-linear relationships inherent in biological data (24, 25). For this, we considered 70% of patient data for training and the rest was used to test and validate the generated models. Surprisingly, these models outreached the predictive value of our biomarkers for fatal outcome, leading to an AUC value of 0.937 (*p* < 0.001, **Figure 6A**). Also, the accuracy of prediction was higher than the one in the training set, 93.8% *vs.* 90.5%. Interestingly sTIM-1, which has been reported as an alternative entry receptor for SARS-CoV-2, exhibited the highest weight in the generated models, surpassing markers identified in previous linear models, such as Gal-9, sCD30 and sCD40. At this point we hypothesized that the applied therapy might have influenced the ultimate outcome, shedding unexpected light on the importance of sTIM-1 in the neural network analysis. Indeed, the presence of a large number of high outliers for sTIM-1 at hospital admission in the survivors’ group was linked to the administration of anti-interleukin therapy (AIT) during hospitalization, either using anti-IL6 monoclonal antibody therapy (Tocilizumab) or an IL-1R antagonist (Anakinra). Importantly, no significant differences in the levels of investigated soluble ICP molecules were detected between the three distinct groups of treatment options: Tocilizumab, Anakinra or non-AIT, and thus, therapy choice was considered in our further analysis (**Supplementary Figures 5**, **6**). Introducing the administered therapy in our neural network models, the importance of sTIM-1 was markedly diminished, falling below that of Gal-9, sCD30 and sCD40, suggesting that AIT improved the final outcome in patients initially presented with high levels of sTIM-1 (**Figure 6B**). Furthermore, incorporating additional confounders into our analysis, such as age, gender, vaccination status, and the presence of various comorbidities, improved even more the prediction capacity (AUC 0.954, *p* < 0.001) and highlighted the significance of sCD163 in discriminating between survivors and non-survivors (**Figure 6C**).

### 3.5 Distinct association patterns of soluble immune checkpoints are linked to different forms of disease severity or SARS-CoV-2 variant infections

**Figure 7:**
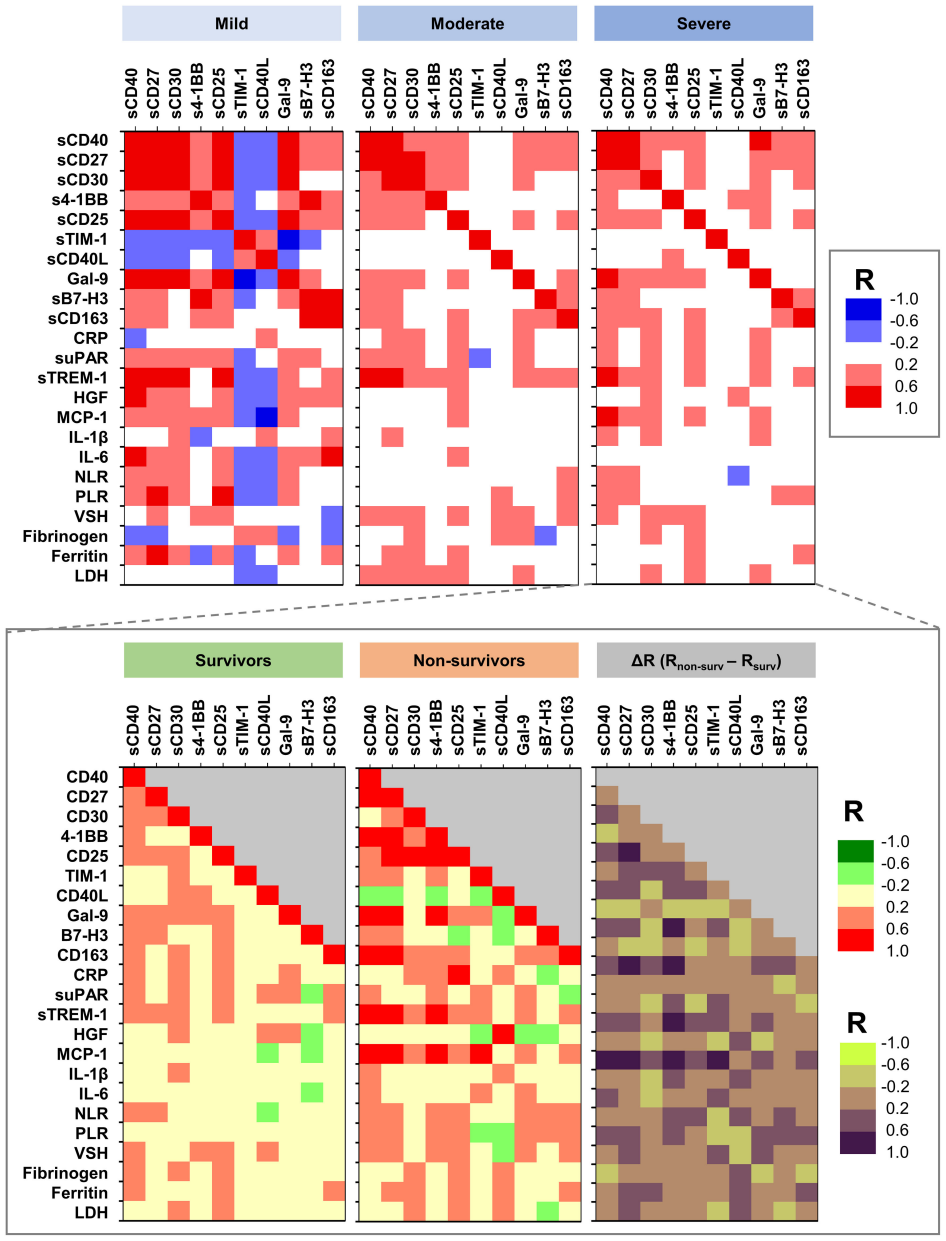
Heat map describing the association between soluble immune checkpoint molecules and previously investigated pro-inflammatory biomarkers in mild, moderate and severe cases of SARS-CoV-2 infection. The graph from the bottom depicts the differences in various associations between survivors and non-survivors (R = correlation coefficient).

At this point, we aimed to further investigate any potential association between soluble immune checkpoint molecules and previously identified pro-inflammatory biomarkers to correlate with disease severity and/or mortality. Interestingly, sTIM-1 and sCD40L negatively associated with the majority of the other soluble immune checkpoint molecules and pro-inflammatory biomarkers in mild cases, while in moderate and severe patients, those negative associations (particularly between sTIM-1 and suPAR, sTREM-1 or MCP-1) diminished and even reversed in the non-survivors’ group (**Figure 7** and **Supplementary Figure 7**). sTREM-1 and MCP-1 also showed very good correlations with sCD40 (R = 0.58, *p* = 0.0118; R = 0.59, *p* = 0.0091), sCD30 (R = 0.33, *p* = 0.1827; R = 0.49, *p* = 0.0379) and s4-1BB (R = 0.61, *p* = 0.0078; R = 0.69, *p* = 0.0014), which further correlated with urea and creatinine levels in the non-survivors’ group (**Supplementary Figure 8**). Importantly, sTIM-1 revealed a strong association with D-dimers (R = 0.62, *p* = 0.0062, **Supplementary Figure 6**). Moreover, strong correlations of sCD40 with sCD27, Gal-9, and suPAR were observed in Omicron infections, while a strong association of sCD40 with sTREM-1 was noticed in Delta infections (**Supplementary Figure 9**). Based on these observations and considering the prognostic values for disease mortality of suPAR, sTREM-1 and MCP-1, we included those biomarkers in the neural network analysis and reached an outstanding model of mortality prediction of AUC 0.977 with suPAR, sTREM-1, sCD30, Galectin-9 and sTIM-1 providing the highest weight in our models (**Supplementary Figure 10**). These results clearly show that neural networks reveal novel determinants of disease severity and mortality and helps in stratifying the importance of various clinical and paraclinical parameters from complex data. Nevertheless, our comprehensive observations indicate that distinct subsets of T cells, B cells and APCs are activated and act differently across varying degrees of disease severity (mild, moderate, severe) or SARS-CoV-2 variant infections (Delta *vs.* Omicron).

### 3.6 Comparison of artificial neural network models with other machine learning algorithms

**Figure 8:**
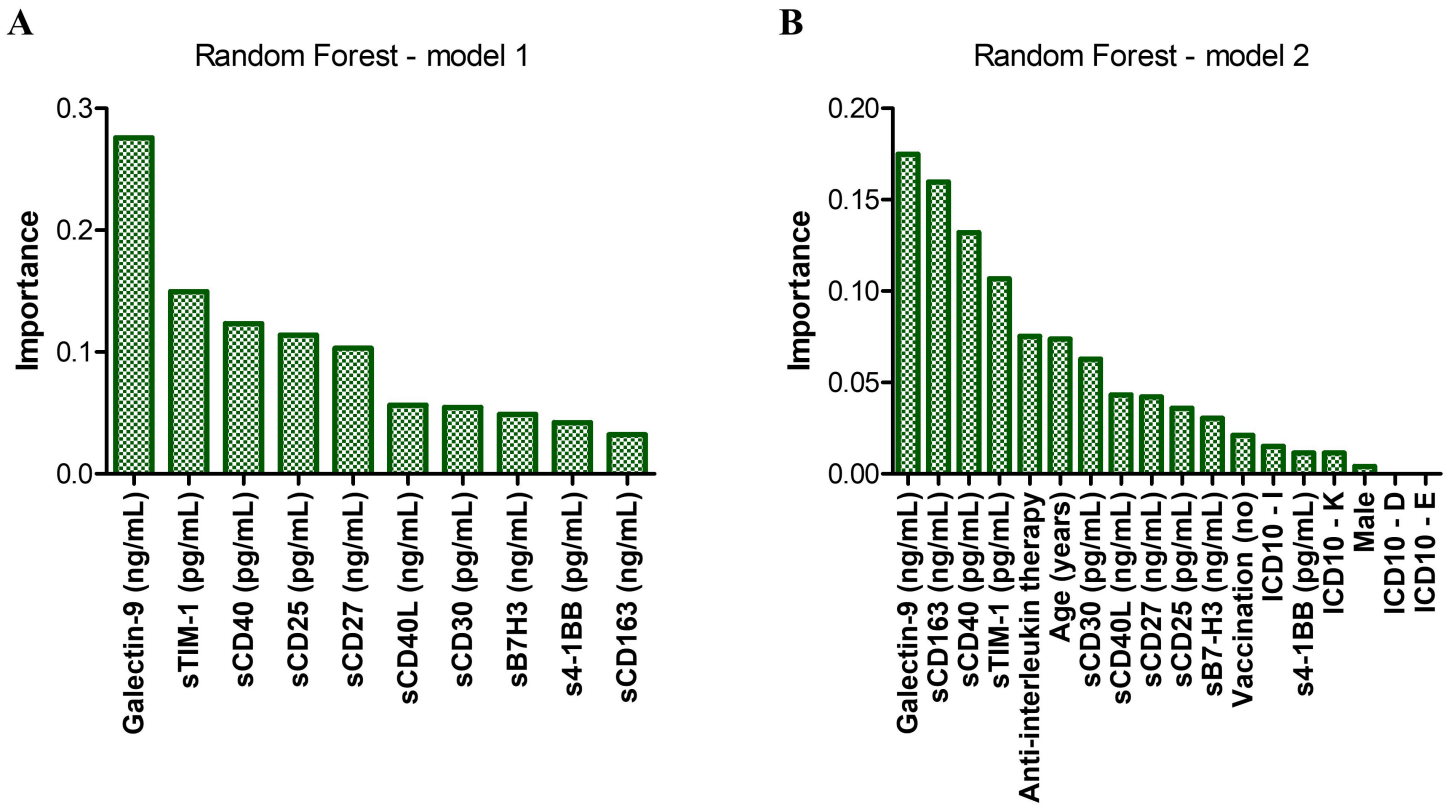
Random forest models of mortality prediction. **(A)** Model 1 – only soluble immune checkpoints included. The accuracy for prediction was 80.4%, while the accuracy for training was 97.1%. **(B)** Model 2 – Model 1 corrected for the therapy received during hospitalization, the presence of comorbidities, vaccination status, age and gender. The accuracy for prediction was 84.7%, while the accuracy for training was 98.1%. Bars represent the importance of each marker in the prediction models.

We next aimed to compare the performance of our generated ANN models with commonly used machine learning algorithms as decision trees (DTs) or random forests (RFs). The RT algorithm works by assembling multiple decision trees to generate a single, more accurate prediction or outcome, and reduce the risk of overfitting. The Random Forest model, developed using Python version 13.2 and based on the initial levels of soluble immune checkpoints, proved a strong predictive accuracy of 80.4% in forecasting patient mortality. This model also validated the significance of the previously identified biomarkers using ANN, particularly Gal-9, sTIM-1, and sCD40, in the context of mortality prediction (**Figure 8A**). Incorporating anti-interleukin therapy and additional clinical data into these models, further validated the importance of sCD163, while the significance of sTIM-1 diminished (**Figure 8B**, prediction accuracy = 84.7%). This adjustment suggests that sCD163 may play a more critical role in determining survival, whereas sTIM-1’s influence is reduced when factoring in the anti-interleukin therapy (Tocilizumab or Anakinra). These observations align with the results obtained from the ANN models, reinforcing the consistency and reliability of our findings across different predictive approaches. Generating DTs using SPSS helped building models of accuracy over 85%. Interestingly, the first model revealed specific cutoff values for key soluble ICPs: 18.57 ng/mL for Gal-9, followed by 3.86 ng/mL for CD40L, 629.36 ng/mL for sCD163 and 84.76 pg/mL for s4-1BB (**Supplementary Figure 11**). Additionally, a favorable response to anti-interleukin therapy was linked to elevated levels of either Gal-9 above 35.42 ng/mL or B7-H3 exceeding 15.7 ng/mL (**Supplementary Figure 12**). Overall, these data validate and complement the ANN findings, underscoring the critical role of soluble immune checkpoints in determining the patient outcome in COVID-19.

## 4 Discussion

Immune checkpoints receptors and ligands are molecules with stimulatory, inhibitory or dual functions in immune cells which maintain the immune surveillance and prevent excessive activation, while ensuring the efficient and effective defense responses against pathogens or cancer cells (14, 26).

Most of the soluble isoforms of immune checkpoints (sICPs) are believed to result from the proteolytical cleavage of membrane-bound proteins or by alternative splicing of mRNA (18) with the role to compete with and counteract the activity of the membrane-bound forms, detailed in **Supplementary Table 11**. The clinical relevance of sICPs remains largely unknown, despite emerging evidence on their dysregulation in patients with viral infections (including the SARS-CoV-2 infection) (17, 20), autoimmune diseases (27) or cancer (18, 28). Resulting from the proteolytical shedding of their membrane bound counterparts upon inflammation, sICPs function, in a first instance, as a mirror of hyperinflammation and activity of metalloproteinases, but also reflect the profile of distinct subsets of immune cells and their checkpoint surface expression. Additionally, while some sICPs maintain the biological activity of their membrane isoforms, others act as molecular decoys or form soluble complexes by interacting with their soluble ligands or receptors (18). Understanding the distinct profile of sICPs associated with various forms of infection is key in decoding the molecular mechanisms of disease pathogenesis which can further provide crucial insights for devising the most effective therapeutic approach.

Here we analyzed the variations in soluble checkpoints receptors and ligands in relation to COVID-19 severity, SARS-CoV-2 variant infection and mortality and compared them to standardized proinflammatory molecules and additional hematological and serological biomarkers. We included in our analysis a list of commonly investigated sICPs (sCD40 with its ligands sCD40L and Gal-9; sCD25; sCD27) as well as molecules newly found and relatively less studied in the literature, such as sCD30, s4-1BB, sTIM-1, sB7-H3 and sCD163. We will continue to discuss the main findings of our study related to each mentioned soluble molecule.

The CD40-CD40L interaction is crucial for immune regulation and activation. This interaction triggers bidirectional signaling of both antigen-presenting cells (APCs, such as dendritic cells, macrophages, B cells) and T cells, leading to upregulation of co-stimulatory molecules and cytokine secretion. Indeed, activation of CD40 on dendritic cells promotes their maturation and enhances their B7 ligands expression, while CD40 signaling in monocytes and macrophages induces the secretion of pro-inflammatory cytokines such as IL-1β and IL-6 (29, 30). CD40 on B cells is of paramount importance for proper activation of B cells and the generation of humoral immune responses. CD40L (also known as CD154) was initially identified on stimulated CD4+ T cells, but now it is known to have a broader immune cell expression, including stimulated CD8+ T cells, mast cells, basophils, and platelets (31, 32). The membrane expression of CD40L on platelets is linked to their activation and further stimulates leukocytes and endothelial cells, by direct engagement of CD40L to CD40 and P selectin to its ligand PGSL-1, to produce proinflammatory cytokines and adhesion molecules (33). Importantly, direct binding of SARS-CoV-2 virions to platelets initiates multiple signaling intracellular pathways, including the AKT-PKC cascade which upregulates the platelet membrane-bound CD40L expression and the release of soluble CD40L molecules stored in platelet granules upon their activation (34, 35). Both soluble isoforms of CD40 and CD40L may also be generated by the shedding of their extracellular domains through the action of specific metalloproteinases such as ADAM10 and ADAM17 (also known as TNF-alpha converting enzyme, TACE) activated upon hyperinflammation conditions (36, 37). Thus, the CD40-CD40L interaction is believed to significantly contribute to monocyte activation, cytokine storm and hypercoagulation associated with severe COVID-19 (29). We here reported increasing levels of sCD40 dependent on disease severity, from mild to moderate and severe COVID-19. In our cohort, sCD40 showed a positive correlation with the pro-inflammatory biomarker IL-6 in mild cases, correlation that diminished in moderate and severe cases, suggesting multiple intricated molecular mechanisms despite increased levels of both biomarkers. Importantly, the non-survivors’ group presented at hospital admission a high association between sCD40 with both MCP-1 and sTREM-1, two well-known biomarkers of monocytes and macrophage overactivation during sepsis and hyperinflammation (3). Interestingly, despite no significant correlation between sCD40L and sCD40 in the non-survivor group, sCD40L showed a high association with HGF and moderate with platelet number and fibrinogen, possibly pointing towards a sepsis hypercoagulable profile characterized by activated platelets releasing higher amounts of both HGF and soluble CD40L. Indeed, this observation is in line with the previously described role of sCD40L as biomarker of unfavorable outcome in patients with severe sepsis or septic shock. These associations in the non-survivors’ group may thus indicate a mechanism of interaction between platelets and monocytes that resulted in the release of high amount of soluble CD40. Additionally, only sCD40 and not sCD40L showed a linear increase pattern from mild to moderate and severe cases (sCD40L did not differentiate between the moderate and severe groups), suggesting additional mechanisms, and possibly, additional cellular contributors in generating the pool of soluble CD40, such as fibroblasts, epithelial and endothelial cells stimulated by hyperinflammatory cytokines characterizing the severe patient cohort.

CD30, another member of the tumor necrosis factor receptor superfamily, is generally strongly expressed in classical Hodgkin lymphoma (cHL) and anaplastic large cell lymphoma, a rare subtype of NHL, with a variable expression observed in T-cell, NK cell or B cell lymphomas (38, 39).

In non-malignant scenarios, CD30 is mainly expressed on a limited subset of activated T cells, B cells, NK cells and regulatory T cells providing pro-survival and anti-apoptotic roles (39, 40). In inflammatory diseases, the extracellular region of the membrane-bound CD30 is easily cleaved by protein hydrolases into soluble fragments (sCD30) (41), thus generally being considered a marker of a subset of activated effector T cells that produce high amount of IFN-γ and IL-5 that exhibit enhanced help activity for B cell Ig production and maintains the Th1/Th2 balance state (42, 43). These CD30+ CD4+ T cells are essential also for sustaining the follicular germinal center responses with the generation of B cells with antigen receptors of high affinity (44). In our cohort, a strong association between sCD30 and CD40 was observed only in mild cases suggesting proper T-B cell responses, while this association reduced in moderate cases and even more in severe patients. However, recent studies have highlighted CD30 as a marker of activated effector regulatory T cells, which are critical for the regulation and/or suppression of memory T and B cells responses (45). These observations are important since, despite a significant increase in the serum levels of both sCD40 and sCD30, sCD30 showed a clear dissociation from sCD40 in non-survivors and a clear association with sCD25, a soluble fragment released from the membrane bound CD25 which is highly expressed on regulatory CD25+ FOXP3+ Tregs, suggesting an increase in the Tregs population within those individuals. These observations are in line with the previous studies reporting a rising percentage of deranged CD25+ FOXP3+ Tregs among CD4+ T cells in severe compared to mild COVID-19 patients which returned to normal levels only in the recovering individuals (4, 11–13, 46). However, compared to sCD30, sCD25 significantly discriminated between mild, moderate and severe patients, suggesting additional cellular sources. For instance, the proinflammatory CD25+ CD8+ T cells with lower cytotoxic activity are expanded in severe COVID-19 patients, being an important source of sCD25 and cause of delayed clearance of virus (9, 47–49). Interestingly, the initial correlation seen between sCD40 and sCD30 in mild cases was replaced among non-survivors, by the association between sCD40 with s4-1BB and sCD27, the soluble forms of the counterpart surface markers of memory CD8+T cells. These observations taken together, indicate a shift of the immune profile towards Tregs and pro-inflammatory CD8+ T cells which suppress the proper T-B cell responses and delays the virus clearance. Indeed, similar to a previous study (17), we confirmed that both s4-1BB and sCD27 were linked to a higher disease severity rate.

Next, we noticed that, in the non-survivors’ group, Gal-9 markedly associated with all sCD40, sCD27 and s4-1BB. Gal-9 is expressed by a variety of immune cells including T and B cells, Tregs, neutrophils, monocytes, macrophages, dendritic cells and are secreted via non-classical pathways or freely released upon cell death with multiple biological functions depending on the interacting receptor CD40, PD-1, TIM-3, 4-1BB (50–52). In a variety of inflammatory conditions and infectious diseases, Gal-9 suppressed the B cell receptor signaling (53) and was commonly associated with exhausted T cells and impaired cytotoxic NK cells (54). Furthermore, Gal-9 through interaction with PD-1 and TIM-3 inhibits T cell proliferation and induces cell-death (55, 56). We, as others (51), found significantly higher levels of Gal-9 in patients with severe disease (ranging from 5.4 ng/mL to 59.7 ng/mL) compared to mild/moderate cases (ranging from 4.1 ng/mL to 23.4 ng/mL) with the highest values observed in the non-survivors’ subgroup (ranging from 19.0 ng/mL to 59.7 ng/mL) and positive association with pro-inflammatory/sepsis biomarkers (sTREM-1, MCP-1, IL-6, NLR, PLR, VSH) and with neutrophils and eosinophils numbers. This last result was expected, since immune activation following SARS-CoV-2 infections causes Gal-9 shedding from neutrophils and eosinophils (51). Interestingly, Treg cells also express high levels of Gal-9 and the Gal-9/Tim-3 signaling pathway further promotes the induction of Tregs, suggesting additional mechanisms for Gal-9 to exhibit suppressive immune responses (57). Additionally, Gal-9 binding to 4-1BB in a region distinct from the binding site of its natural ligand (4-1BBL), augments 4-1BB signaling and activity in T cells and NK cells to produce proinflammatory molecules (TNF-α, MCP-1) Thus, the Gal-9 induced release of TNF-α and MCP-1 activates metalloproteinases (e.g., ADAM10, ADAM17) which further mediates the ectodomain shedding of 4-1BB from T cells (58) and the scavenger receptor CD163, who’s expression is induced by the immunosuppressive IL-10 on the membrane of M2 macrophages, cells with anti-inflammatory activity (59). Indeed, s4-1BB showed strong associations with MCP-1 and sCD163 at baseline in the non-survivors’ group from our study. However, shedding of 4-1BB further limits the Gal-9 pro-inflammatory activity (58). These observations are important, since the suppressive immune activity of both Gal-9 and sCD163 in association with high sCD40 basal levels were mainly revealed by the neural network models corrected for the therapeutic strategy, age, vaccination status and the presence of comorbidities.

Further, the neural network analysis identified TIM-1 to bring the highest contribution among the investigated checkpoint molecules in discriminating between survivors and non-survivors. Our analysis further revealed important correlations between sTIM-1 with sCD40, MCP-1, Gal-9, sTREM-1 and suPAR. Among non-immune cells, TIM-1 glycoprotein is expressed by lung and kidney epithelial cells where it serves as a phosphatidylserine receptor and thus, as an alternative receptor to ACE-2 for SARS-CoV-2 (60). The soluble form is released from the membrane bound TIM-1 by proteolytical cleavage mediated by ADM-10 and ADAM-17 (61).

Not surprisingly, sTIM-1 and sCD40L strongly correlated in mild cases of infections, since SARS-CoV-2 modulates their cellular expression of infected epithelial cells and platelets (34, 35). However, this correlation was inversed in the non-survivors’ group, possibly indicating the existence of additional cellular sources for sTIM-1, including immune cells such as Th2 cells, NK cells, and regulatory B cells within these individuals (62, 63). Since sTIM-1 still binds phosphatidylserine, it may serve as a negative regulator of cellular TIM-1 (61). For instance, TIM-1 mediates T cell trafficking by directly binding the adhesion receptor P-selectin during inflammation (64). Blocking TIM-1 signaling in B cells enhances the type 1 interferon response within these cells, leading to increased B cell activation and antigen presentation, along with heightened co-stimulation capabilities (62, 63). Importantly, in our patient cohort, all the cases with initially high sTIM-1 levels (over 300 pg/mL) who received specific anti-inflammatory therapy (Tocilizumab or Anakinra) survived, pointing towards the beneficial effect of this therapy in influencing the final outcome of selected patients. The proposed ANN and Random Forest models highlighted the role of soluble TIM-1 and Gal-9 in predicting mortality among COVID-19 patients, effect that was diminished when anti-interleukin therapy was added into these models. This finding suggests that the administration of Tocilizumab or Anakinra may have been particularly beneficial for patients with initially elevated soluble sTIM-1 and Gal-9 levels, increasing their chance of survival. This observation becomes even more relevant when considering the strong association between sTIM-1 and D-dimers, indicating a heightened risk for coagulopathy or thrombotic events in patients with initially elevated sTIM-1 levels. Thus, high sTIM-1 and Gal-9 levels may reflect an intensified inflammatory response, and by modulating the immune response, this targeted therapy could help reduce inflammation, leading to better clinical outcomes for these patients. When comparing the Delta and Omicron variant infections, few differences in the checkpoint ligands’ profile emerged. First, a reduction in the sCD40L and Gal9 levels were noticed in the Omicron variant group, which was associated with a lower mortality rate among affected individuals. Alongside this decrease, there was a concurrent rise in sB7-H3 levels, a newly investigated molecule in literature. sB7-H3 is a functionally active form that is released by the ectodomain shedding of membrane-bound CD276 (mB7-H3) from the surface of monocytes, dendritic cells and activated T cells by various metalloproteinase (65). B7-H3 has been described as a checkpoint molecule with dual function, serving both as a stimulatory factor for T cell activation and IFN-γ production, as well as exhibiting inhibitory effects on T cell proliferation and Th1 differentiation (66–68). This is important since in our analysis, sB7-H3 appeared to play a primarily inhibitory role by limiting overactivation and/or exhaustion of T cells in individuals infected with the Omicron-variant.

Our analysis is however limited to a relatively small sample size and the focus on only ten distinct soluble immune checkpoint molecules. This highlights the need for future studies to cross-validate or ensemble these models on larger and/or different datasets, which could further enhance prediction accuracy and reduce the risk of overfitting. With the aim of exploring a series of less studies ICPs, this study primarily focused on examining stimulatory checkpoint molecules, while including only few inhibitory ones, specifically sTIM-1 and Gal-9. Future studies should also incorporate more well-known inhibitory molecules, such as LAG-3, GITR, PD-1, and CTLA-4, whose alterations are commonly associated with viral infections and COVID-19, such as LAG-3, GITR, PD-1, or CTLA-4 (69). For instance, LAG-3 expressed primarily on exhausted CD8+ T cells, acts by inhibiting T cell proliferation and cytokine production, thereby acting as a compensatory mechanism to excessive immune activation. Elevated levels of soluble LAG-3, along with sTIM-3, s-GITR, sPD1, and sCTLA-4 were consistently higher in patients with severe and critical COVID-19, and these levels were negatively correlated with the absolute counts of CD4+ and CD8+ T cells (17, 19). Importantly, Gal-9, the ligand of TIM-3 mirrored these findings, further validating the importance of our study.

## 5 Conclusions

**Figure 9:**
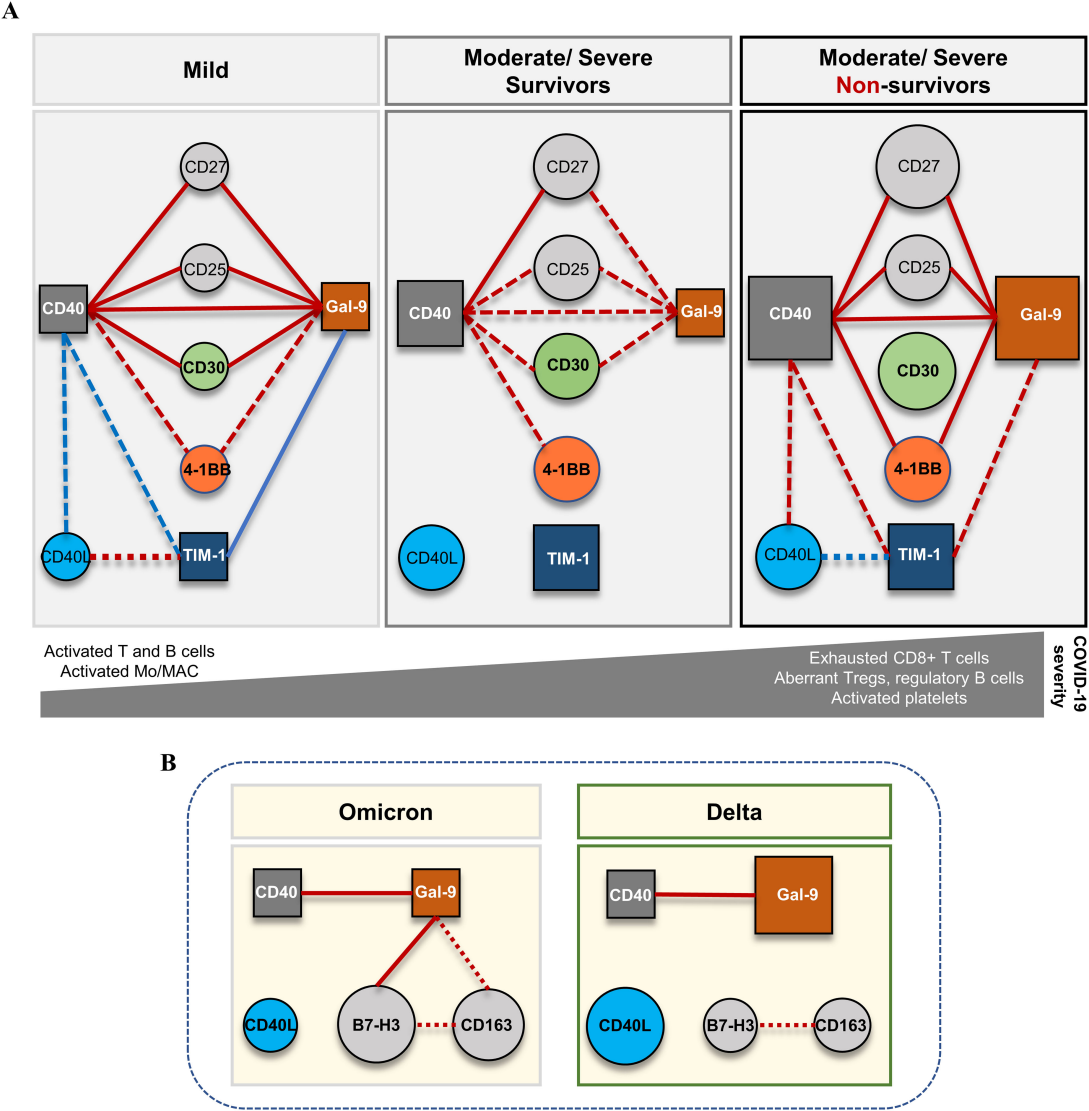
Schematic representation of the proposed model for the dynamics of soluble immune checkpoints in COVID-19. **(A)** The network model based on the serum levels of soluble immune checkpoint molecules and their interconnected associations in different forms of disease severity. Soluble isoforms of CD40, Gal-9 and TIM-1 are key players interconnecting the soluble variants of co-stimulatory immune checkpoint receptors CD27, CD25, CD30, 4-1BB. The profile of soluble immune checkpoint molecules in mild cases is suggestive for efficient cooperation of T and B cell responses and proper antigen presentation cell (APC) functioning. **(B)** The network model based on the serum levels of soluble immune checkpoint molecules and their interconnected associations in different forms of variant infection: Delta *vs.* Omicron. Soluble forms of CD40 and Gal-9 also play central role and interconnect to soluble B7-H3 and CD163 molecules. Red lines indicate positive associations, while blue lines indicate negative associations with dot lines designated for moderate correlations and full lines for strong correlations. The size of circles and squares reflects the relative median values of represented soluble biomarkers for each category of patients.

This study is of importance as it provides baseline values for a series of soluble immune checkpoint receptors and ligands for which only limited data exist in literature, and identify the biomarkers associated with disease severity and mortality in COVID-19. For instance, our study confirms the association of Gal-9 and sCD25 with disease severity and identifies sCD40 to play a central role in defining the distinct immune profiles characterizing the patients with mild, moderate and severe COVID-19 at hospital admission (see the diagram in **Figure 9**). Our study also points out the differences in the serum profiles of soluble immune checkpoint ligands between Delta and Omicron variant infections, and identifies associations of sCD40L and Gal-9 with sB7-H3 and sCD163 levels in subjects infected with the Omicron variant (**Figure 9**). Despite the limitation of not offering information in dynamics, our study further provides correlations and interaction models with previously documented hyperinflammatory molecules (suPAR, sTREM-1, HGF, MCP-1, IL-1β, IL-6, ferritin) (3), alongside hematological and coagulation profiles (revealed by NLR, PLR, ESR, fibrinogen, D-dimer), markers of multiple organ injury (LDH, creatinine, urea, AST, ALT, total bilirubin) and data regarding therapeutic strategies, comorbidities, and vaccination status. Nevertheless, this study also points out the importance of including neural network models in complex data analysis for identifying the key elements and molecular-therapeutic interactions that underlines the overall paraclinical and clinical patient’s profile.

## Data Availability

The original contributions presented in the study are included in the article/**Supplementary Material**. Further inquiries can be directed to the corresponding author.

## References

[1] Xu Z Shi L Wang Y Zhang J Huang L Zhang C. Pathological findings of COVID-19 associated with acute respiratory distress syndrome. Lancet Respir Med. (2020) 8:420–2. doi: 10.1016/S2213-2600(20)30076-X PMC7164771 32085846

[2] Weiss P Murdoch DR. Clinical course and mortality risk of severe COVID-19. Lancet. (2020) 395:1014–5. doi: 10.1016/S0140-6736(20)30633-4 PMC7138151 32197108

[3] Paranga TG Pavel-Tanasa M Constantinescu D Plesca CE Petrovici C Miftode IL. Comparison of C-reactive protein with distinct hyperinflammatory biomarkers in association with COVID-19 severity, mortality and SARS-CoV-2 variants. Front Immunol. (2023) 14:1213246. doi: 10.3389/fimmu.2023.1213246 37388734 PMC10302717

[4] Dhawan M Rabaan AA Alwarthan S Alhajri M Halwani MA Alshengeti A. Regulatory T cells (Tregs) and COVID-19: unveiling the mechanisms, and therapeutic potentialities with a special focus on long COVID. Vaccines (Basel). (2023) 11:699. doi: 10.3390/vaccines11030699 36992283 PMC10059134

[5] Mathew D Giles JR Baxter AE Oldridge DA Greenplate AR Wu JE. Deep immune profiling of COVID-19 patients reveals distinct immunotypes with therapeutic implications. Science. (2020) 369:eabc8511. doi: 10.1126/science.abc8511 32669297 PMC7402624

[6] Grau-Exposito J Sanchez-Gaona N Massana N Suppi M Astorga-Gamaza A Perea D. Peripheral and lung resident memory T cell responses against SARS-CoV-2. Nat Commun. (2021) 12:3010. doi: 10.1038/s41467-021-23333-3 34021148 PMC8140108

[7] Maison DP Deng Y Gerschenson M. SARS-CoV-2 and the host-immune response. Front Immunol. (2023) 14:1195871. doi: 10.3389/fimmu.2023.1195871 37404823 PMC10315470

[8] Sekine T Perez-Potti A Rivera-Ballesteros O Stralin K Gorin JB Olsson A. Robust T cell immunity in convalescent individuals with asymptomatic or mild COVID-19. Cell. (2020) 183:158–68.e14. doi: 10.1016/j.cell.2020.08.017 32979941 PMC7427556

[9] Mazzoni A Salvati L Maggi L Capone M Vanni A Spinicci M. Impaired immune cell cytotoxicity in severe COVID-19 is IL-6 dependent. J Clin Invest. (2020) 130:4694–703. doi: 10.1172/JCI138554 PMC7456250 32463803

[10] Zheng M Gao Y Wang G Song G Liu S Sun D. Functional exhaustion of antiviral lymphocytes in COVID-19 patients. Cell Mol Immunol. (2020) 17:533–5. doi: 10.1038/s41423-020-0402-2 PMC7091858 32203188

[11] Neumann J Prezzemolo T Vanderbeke L Roca CP Gerbaux M Janssens S. Humblet-Baron: Increased IL-10-producing regulatory T cells are characteristic of severe cases of COVID-19. Clin Transl Immunol. (2020) 9:e1204. doi: 10.1002/cti2.1204 PMC7662088 33209300

[12] Galvan-Pena S Leon J Chowdhary K Michelson DA Vijaykumar B Yang L. Profound Treg perturbations correlate with COVID-19 severity. Proc Natl Acad Sci U S A. (2021) 118:e2111315118. doi: 10.1073/pnas.2111315118 34433692 PMC8449354

[13] Nam H Koh JY Jung JH Jeong H Jeong HW Cheon S. Distinctive dynamics and functions of the CD4+CD25+FOXP3+ Regulatory T cell population in patients with severe and mild COVID-19. J Immunol. (2023) 210:1687–99. doi: 10.4049/jimmunol.2200290 37042681

[14] Chen L Flies DB. Molecular mechanisms of T cell co-stimulation and co-inhibition. Nat Rev Immunol. (2013) 13:227–42. doi: 10.1038/nri3405 PMC3786574 23470321

[15] Wei SC Duffy CR Allison JP. Fundamental mechanisms of immune checkpoint blockade therapy. Cancer Discovery. (2018) 8:1069–86. doi: 10.1158/2159-8290.CD-18-0367 30115704

[16] Tan J Li Y. Immune checkpoint alterations and their blockade in COVID-19 patients. Blood Sci. (2022) 4:192–8. doi: 10.1097/BS9.0000000000000132 PMC9592141 36311817

[17] Kong Y Wang Y Wu X Han J Li G Hua M. Storm of soluble immune checkpoints associated with disease severity of COVID-19. Signal Transduct Target Ther. (2020) 5:192. doi: 10.1038/s41392-020-00308-2 32895366 PMC7475713

[18] Gu D Ao X Yang Y Chen Z Xu X. Soluble immune checkpoints in cancer: production, function and biological significance. J Immunother Cancer. (2018) 6:132. doi: 10.1186/s40425-018-0449-0 30482248 PMC6260693

[19] Avendano-Ortiz J Lozano-Rodriguez R Martin-Quiros A Terron V Maroun-Eid C Montalban-Hernandez K. The immune checkpoints storm in COVID-19: Role as severity markers at emergency department admission. Clin Transl Med. (2021) 11:e573. doi: 10.1002/ctm2.573 34709745 PMC8521292

[20] Li W Syed F Yu R Yang J Xia Y Relich RF. Soluble immune checkpoints are dysregulated in COVID-19 and heavy alcohol users with HIV infection. Front Immunol. (2022) 13:833310. doi: 10.3389/fimmu.2022.833310 35281051 PMC8904355

[21] Pavel-Tanasa M Constantinescu D Cianga CM Anisie E Mereuta AI Tuchilus CG. Adipokines, and not vitamin D, associate with antibody immune responses following dual BNT162b2 vaccination within individuals younger than 60 years. Front Immunol. (2022) 13:1000006. doi: 10.3389/fimmu.2022.1000006 36119038 PMC9481237

[22] Kapsiani S Howlin BJ. Random forest classification for predicting lifespan-extending chemical compounds. Sci Rep. (2021) 11:13812. doi: 10.1038/s41598-021-93070-6 34226569 PMC8257600

[23] Martinez-Diz S Marin-Benesiu F Lopez-Torres G Santiago O Diaz-Cuellar JF Martin-Esteban S. Relevance of TMPRSS2, CD163/CD206, and CD33 in clinical severity stratification of COVID-19. Front Immunol. (2022) 13:1094644. doi: 10.3389/fimmu.2022.1094644 36969980 PMC10031647

[24] Hartman E Scott AM Karlsson C Mohanty T Vaara ST Linder A. Interpreting biologically informed neural networks for enhanced proteomic biomarker discovery and pathway analysis. Nat Commun. (2023) 14:5359. doi: 10.1038/s41467-023-41146-4 37660105 PMC10475049

[25] Zhang Z Beck MW Winkler DA Huang B Sibanda W Goyal H. Opening the black box of neural networks: methods for interpreting neural network models in clinical applications. Ann Transl Med. (2018) 6:216. doi: 10.21037/atm.2018.05.32 30023379 PMC6035992

[26] Sharma P Allison JP. The future of immune checkpoint therapy. Science. (2015) 348:56–61. doi: 10.1126/science.aaa8172 25838373

[27] Pyo JY Yoon T Ahn SS Song JJ Park YB Lee SW. Soluble immune checkpoint molecules in patients with antineutrophil cytoplasmic antibody-associated vasculitis. Sci Rep. (2022) 12:21319. doi: 10.1038/s41598-022-25466-x 36494415 PMC9734661

[28] Zhu X Lang J. Soluble PD-1 and PD-L1: predictive and prognostic significance in cancer. Oncotarget. (2017) 8:97671–82. doi: 10.18632/oncotarget.18311 PMC5722594 29228642

[29] Kiener PA Moran-Davis P Rankin BM Wahl AF Aruffo A Hollenbaugh D. Stimulation of CD40 with purified soluble gp39 induces proinflammatory responses in human monocytes. J Immunol. (1995) 155:4917–25. doi: 10.4049/jimmunol.155.10.4917 7594496

[30] Ma DY Clark EA. The role of CD40 and CD154/CD40L in dendritic cells. Semin Immunol. (2009) 21:265–72. doi: 10.1016/j.smim.2009.05.010 PMC2749083 19524453

[31] Elgueta R Benson MJ de Vries VC Wasiuk A Guo Y Noelle RJ. Molecular mechanism and function of CD40/CD40L engagement in the immune system. Immunol Rev. (2009) 229:152–72. doi: 10.1111/j.1600-065X.2009.00782.x PMC3826168 19426221

[32] Elizondo DM Andargie TE Kubhar DS Gugssa A Lipscomb MW. CD40-CD40L cross-talk drives fascin expression in dendritic cells for efficient antigen presentation to CD4+ T cells. Int Immunol. (2017) 29:121–31. doi: 10.1093/intimm/dxx013 PMC5421612 28369442

[33] Yokoyama S Ikeda H Haramaki N Yasukawa H Murohara T Imaizumi T. Platelet P-selectin plays an important role in arterial thrombogenesis by forming large stable platelet-leukocyte aggregates. J Am Coll Cardiol. (2005) 45:1280–6. doi: 10.1016/j.jacc.2004.12.071 15837262

[34] Li T Yang Y Li Y Wang Z Ma F Luo R. Platelets mediate inflammatory monocyte activation by SARS-CoV-2 spike protein. J Clin Invest. (2022) 132:e150101. doi: 10.1172/JCI150101 34964720 PMC8843740

[35] Valet C Magnen M Qiu L Cleary SJ Wang KM Ranucci S. Sepsis promotes splenic production of a protective platelet pool with high CD40 ligand expression. J Clin Invest. (2022) 132:e153920. doi: 10.1172/JCI153920 35192546 PMC8970674

[36] Klersy A Meyer S Leuschner F Kessler T Hecker M Wagner AH. Ectodomain shedding by ADAM17 increases the release of soluble CD40 from human endothelial cells under pro-inflammatory conditions. Cells. (2023) 12:1926. doi: 10.3390/cells12151926 37566005 PMC10417149

[37] Maurer S Kopp HG Salih HR Kropp KN. Modulation of immune responses by platelet-derived ADAM10. Front Immunol. (2020) 11:44. doi: 10.3389/fimmu.2020.00044 32117229 PMC7012935

[38] Nakashima M Uchimaru K. CD30 expression and its functions during the disease progression of adult T-cell leukemia/lymphoma. Int J Mol Sci. (2023) 24:8731. doi: 10.3390/ijms24108731 37240076 PMC10218159

[39] Li Z Guo W Bai O. Mechanism of action and therapeutic targeting of CD30 molecule in lymphomas. Front Oncol. (2023) 13:1301437. doi: 10.3389/fonc.2023.1301437 38188299 PMC10767573

[40] Cui D Zhang Y Chen L Du H Zheng B Huang M. CD30 plays a role in T-dependent immune response and T cell proliferation. FASEB J. (2024) 38:e23365. doi: 10.1096/fj.202301747RR 38069862

[41] Faber ML Oldham RAA Thakur A Rademacher MJ Kubicka E Dlugi TA. Novel anti-CD30/CD3 bispecific antibodies activate human T cells and mediate potent anti-tumor activity. Front Immunol. (2023) 14:1225610. doi: 10.3389/fimmu.2023.1225610 37646042 PMC10461807

[42] Pellegrini P Berghella AM Contasta I Adorno D. CD30 antigen: not a physiological marker for TH2 cells but an important costimulator molecule in the regulation of the balance between TH1/TH2 response. Transpl Immunol. (2003) 12:49–61. doi: 10.1016/S0966-3274(03)00014-5 14551032

[43] Alzona M Jack HM Fisher RI Ellis TM. CD30 defines a subset of activated human T cells that produce IFN-gamma and IL-5 and exhibit enhanced B cell helper activity. J Immunol. (1994) 153:2861–7. doi: 10.4049/jimmunol.153.7.2861 8089475

[44] Gaspal FM Kim MY McConnell FM Raykundalia C Bekiaris V Lane PJ. Mice deficient in OX40 and CD30 signals lack memory antibody responses because of deficient CD4 T cell memory. J Immunol. (2005) 174:3891–6. doi: 10.4049/jimmunol.174.7.3891 15778343

[45] Dai Z Li Q Wang Y Gao G Diggs LS Tellides G. CD4+CD25+ regulatory T cells suppress allograft rejection mediated by memory CD8+ T cells via a CD30-dependent mechanism. J Clin Invest. (2004) 113:310–7. doi: 10.1172/JCI19727 PMC311434 14722622

[46] Vick SC Frutoso M Mair F Konecny AJ Greene E Wolf CR. A regulatory T cell signature distinguishes the immune landscape of COVID-19 patients from those with other respiratory infections. Sci Adv. (2021) 7:eabj0274. doi: 10.1126/sciadv.abj0274 34757794 PMC8580318

[47] Xie M Yunis J Yao Y Shi J Yang Y Zhou P. High levels of soluble CD25 in COVID-19 severity suggest a divergence between anti-viral and pro-inflammatory T-cell responses. Clin Transl Immunol. (2021) 10:e1251. doi: 10.1002/cti2.1251 PMC7883478 33614032

[48] Chen Z John Wherry E. T cell responses in patients with COVID-19. Nat Rev Immunol. (2020) 20:529–36. doi: 10.1038/s41577-020-0402-6 PMC7389156 32728222

[49] Zheng HY Zhang M Yang CX Zhang N Wang XC Yang XP. Elevated exhaustion levels and reduced functional diversity of T cells in peripheral blood may predict severe progression in COVID-19 patients. Cell Mol Immunol. (2020) 17:541–3. doi: 10.1038/s41423-020-0401-3 PMC7091621 32203186

[50] Iwasaki-Hozumi H Chagan-Yasutan H Ashino Y Hattori T. Blood levels of galectin-9, an immuno-regulating molecule, reflect the severity for the acute and chronic infectious diseases. Biomolecules. (2021) 11:430. doi: 10.3390/biom11030430 33804076 PMC7998537

[51] Bozorgmehr N Mashhouri S Perez Rosero E Xu L Shahbaz S Sligl W. Galectin-9, a player in cytokine release syndrome and a surrogate diagnostic biomarker in SARS-CoV-2 infection. mBio. (2021) 12:e00384-21. doi: 10.1128/mBio.00384-21 33947753 PMC8262904

[52] Shahbaz S Dunsmore G Koleva P Xu L Houston S Elahi S. Galectin-9 and VISTA expression define terminally exhausted T cells in HIV-1 infection. J Immunol. (2020) 204:2474–91. doi: 10.4049/jimmunol.1901481 32205423

[53] Giovannone N Liang J Antonopoulos A Geddes Sweeney J King SL Pochebit SM. Galectin-9 suppresses B cell receptor signaling and is regulated by I-branching of N-glycans. Nat Commun. (2018) 9:3287. doi: 10.1038/s41467-018-05770-9 30120234 PMC6098069

[54] Madireddi S Eun SY Lee SW Nemcovicova I Mehta AK Zajonc DM. Galectin-9 controls the therapeutic activity of 4-1BB-targeting antibodies. J Exp Med. (2014) 211:1433–48. doi: 10.1084/jem.20132687 PMC4076583 24958847

[55] Yang R Sun L Li CF Wang YH Yao J Li H. Galectin-9 interacts with PD-1 and TIM-3 to regulate T cell death and is a target for cancer immunotherapy. Nat Commun. (2021) 12:832. doi: 10.1038/s41467-021-21099-2 33547304 PMC7864927

[56] Kanzaki M Wada J Sugiyama K Nakatsuka A Teshigawara S Murakami K. Galectin-9 and T cell immunoglobulin mucin-3 pathway is a therapeutic target for type 1 diabetes. Endocrinology. (2012) 153:612–20. doi: 10.1210/en.2011-1579 22186414

[57] Pang N Alimu X Chen R Muhashi M Ma J Chen G. Activated Galectin-9/Tim3 promotes Treg and suppresses Th1 effector function in chronic lymphocytic leukemia. FASEB J. (2021) 35:e21556. doi: 10.1096/fj.202100013R 34137463

[58] Nielsen MA Andersen T Etzerodt A Kragstrup TW Rasmussen TK Stengaard-Pedersen K. A disintegrin and metalloprotease-17 and galectin-9 are important regulators of local 4-1BB activity and disease outcome in rheumatoid arthritis. Rheumatol (Oxford). (2016) 55:1871–9. doi: 10.1093/rheumatology/kew237 27330157

[59] Etzerodt A Maniecki MB Moller K Moller HJ Moestrup SK. Tumor necrosis factor alpha-converting enzyme (TACE/ADAM17) mediates ectodomain shedding of the scavenger receptor CD163. J Leukoc Biol. (2010) 88:1201–5. doi: 10.1189/jlb.0410235 20807704

[60] Mori Y Fink C Ichimura T Sako K Mori M Lee NN. KIM-1/TIM-1 is a receptor for SARS-CoV-2 in lung and kidney. medRxiv. (2022). doi: 10.1101/2020.09.16.20190694

[61] Schweigert O Dewitz C Moller-Hackbarth K Trad A Garbers C Rose-John S. Soluble T cell immunoglobulin and mucin domain (TIM)-1 and -4 generated by A Disintegrin And Metalloprotease (ADAM)-10 and -17 bind to phosphatidylserine. Biochim Biophys Acta. (2014) 1843:275–87. doi: 10.1016/j.bbamcr.2013.11.014 24286866

[62] Nakae S Iikura M Suto H Akiba H Umetsu DT Dekruyff RH. TIM-1 and TIM-3 enhancement of Th2 cytokine production by mast cells. Blood. (2007) 110:2565–8. doi: 10.1182/blood-2006-11-058800 PMC1988955 17620455

[63] Bod L Kye YC Shi J Torlai Triglia E Schnell A Fessler J. B-cell-specific checkpoint molecules that regulate anti-tumor immunity. Nature. (2023) 619:348–56. doi: 10.1038/s41586-023-06231-0 PMC10795478 37344597

[64] Angiari S Donnarumma T Rossi B Dusi S Pietronigro E Zenaro E. TIM-1 glycoprotein binds the adhesion receptor P-selectin and mediates T cell trafficking during inflammation and autoimmunity. Immunity. (2014) 40:542–53. doi: 10.1016/j.immuni.2014.03.004 PMC4066214 24703780

[65] Zhang G Hou J Shi J Yu G Lu B Zhang X. Soluble CD276 (B7-H3) is released from monocytes, dendritic cells and activated T cells and is detectable in normal human serum. Immunology. (2008) 123:538–46. doi: 10.1111/j.1365-2567.2007.02723.x PMC2433324 18194267

[66] Chapoval AI Ni J Lau JS Wilcox RA Flies DB Liu D. B7-H3: a costimulatory molecule for T cell activation and IFN-gamma production. Nat Immunol. (2001) 2:269–74. doi: 10.1038/85339 11224528

[67] Suh WK Gajewska BU Okada H Gronski MA Bertram EM Dawicki W. The B7 family member B7-H3 preferentially down-regulates T helper type 1-mediated immune responses. Nat Immunol. (2003) 4:899–906. doi: 10.1038/ni967 12925852

[68] Antohe I Tanasa MP Dascalescu A Danaila C Titieanu A Zlei M. The MHC-II antigen presentation machinery and B7 checkpoint ligands display distinctive patterns correlated with acute myeloid leukemias blast cells HLA-DR expression. Immunobiology. (2021) 226:152049. doi: 10.1016/j.imbio.2020.152049 33352400

[69] Cai H Liu G Zhong J Zheng K Xiao H Li C. Immune checkpoints in viral infections. Viruses. (2020) 12:1051. doi: 10.3390/v12091051 32967229 PMC7551039

