# Supplementary material for: Distinct soluble immune checkpoint profiles characterize COVID-19 severity, mortality and SARS-CoV-2 variant infections
Source: Front Immunol. 2024 Sep 23;15:1464480. doi: 10.3389/fimmu.2024.1464480 (PMC11456479; doi:10.3389/fimmu.2024.1464480)
Supplement: Supplementary file 1 [file DataSheet1.pdf]

Distinct soluble immune checkpoint profiles characterize COVID-19 severity, mortality and SARS-CoV-2 variant infections

Tudorita Gabriela Paranga, Mariana Pavel-Tanasa*, Daniela Constantinescu, Elena Iftimi, Claudia Elena Plesca, Ionela-Larisa Miftode, Petru Cianga, Egidia Miftode

*** Correspondence:**
Mariana Pavel-Tanasa, mariana.pavel-tanasa@umfiasi.ro

Supplementary materials contain:

Supplementary Figures 1-12
Supplementary Tables 1-11

Supplementary Figure 1

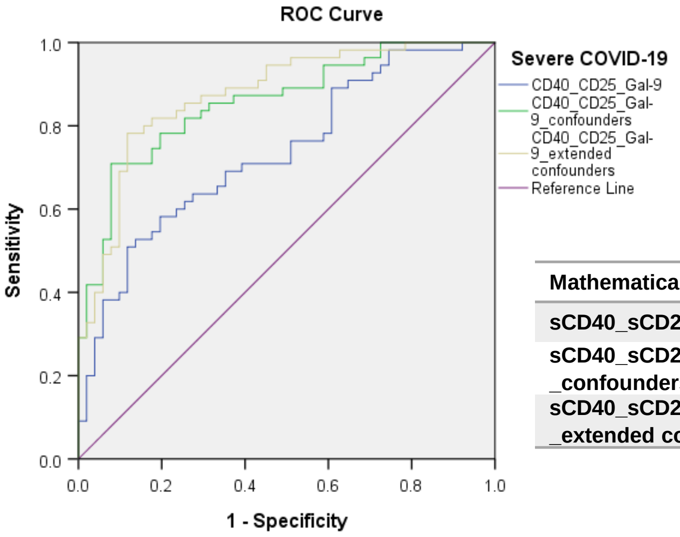

Mathematical model	AUC	S.E.M.	*p*-value	95% CI
**sCD40_sCD25_Gal-9**	0.735	0.048	< 0.001	0.641-0.829
**sCD40_sCD25_Gal-9 _confounders**	0.856	0.036	< 0.001	0.785-0.926
**sCD40_sCD25_Gal-9 _extended confounders**	0.875	0.034	< 0.001	0.809-0.941

Supplementary Figure 1. ROC curves resulted from the models comprising the positive determinants of severe infection and corrected for confounders. General confounders are described in Supplementary Table 4, while extended confounders additionally include all patients characteristics described in Supplementary Table 3. The AUC values between 0.7-0.8 define a very good discrimination, while the AUC values > 0.8 denote an excellent capacity of prediction.

Supplementary Figure 2

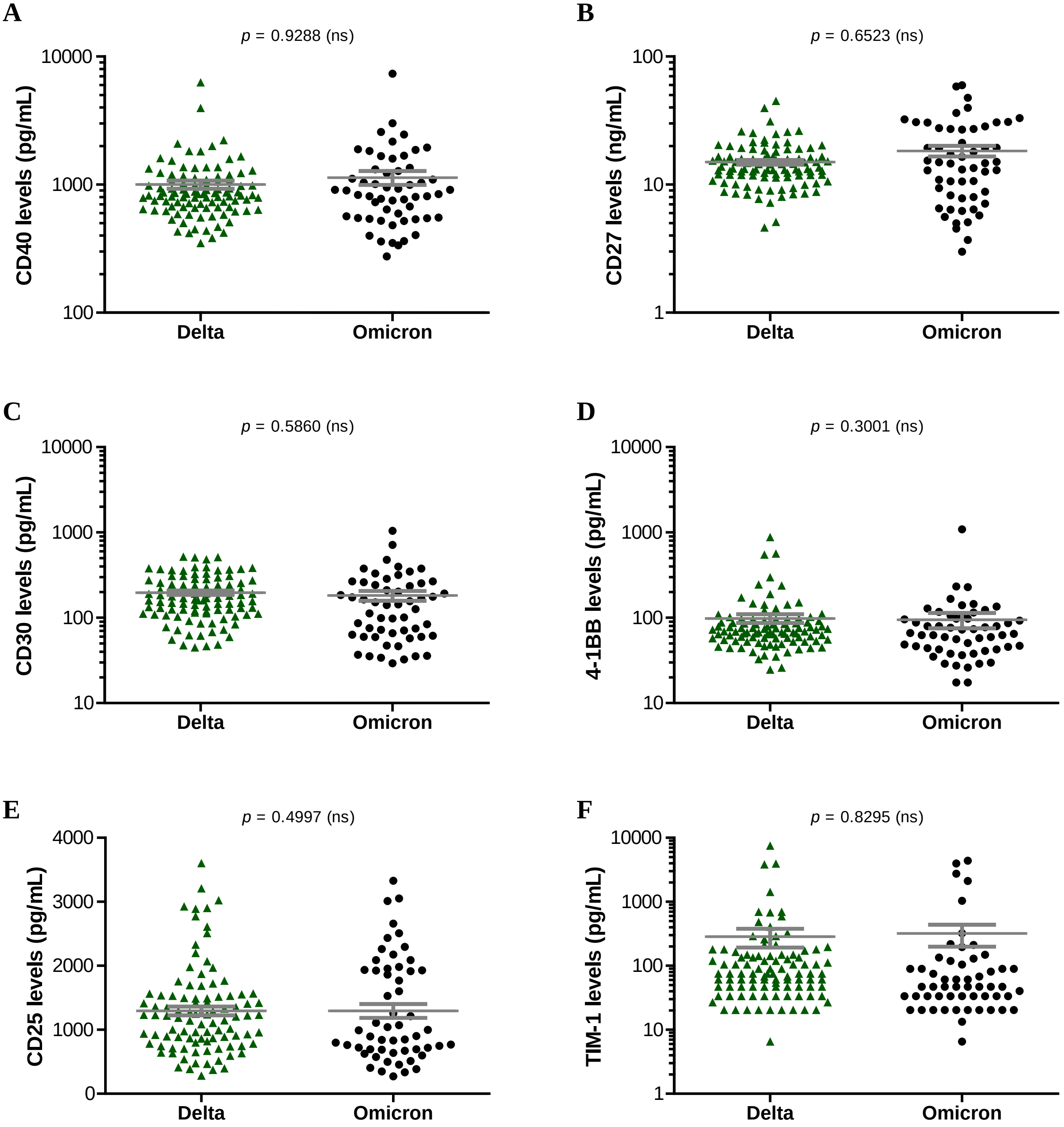

Supplementary Figure 2. Serum profile of soluble immune checkpoint receptors in Delta and Omicron SARS-CoV-2 infections. Serum levels of **(A)** sCD40, **(B)** sCD27, **(C)** sCD30, **(D)** s4-1BB, **(E)** sCD25, **(F)** sTIM-1 for each category of SARS-CoV-2 infection: Delta or Omicron. The gray lines represent the mean ± SEM (ns – not significant; Kruskal-Wallis with Dunn's Multiple Comparison test).

Supplementary Figure 3

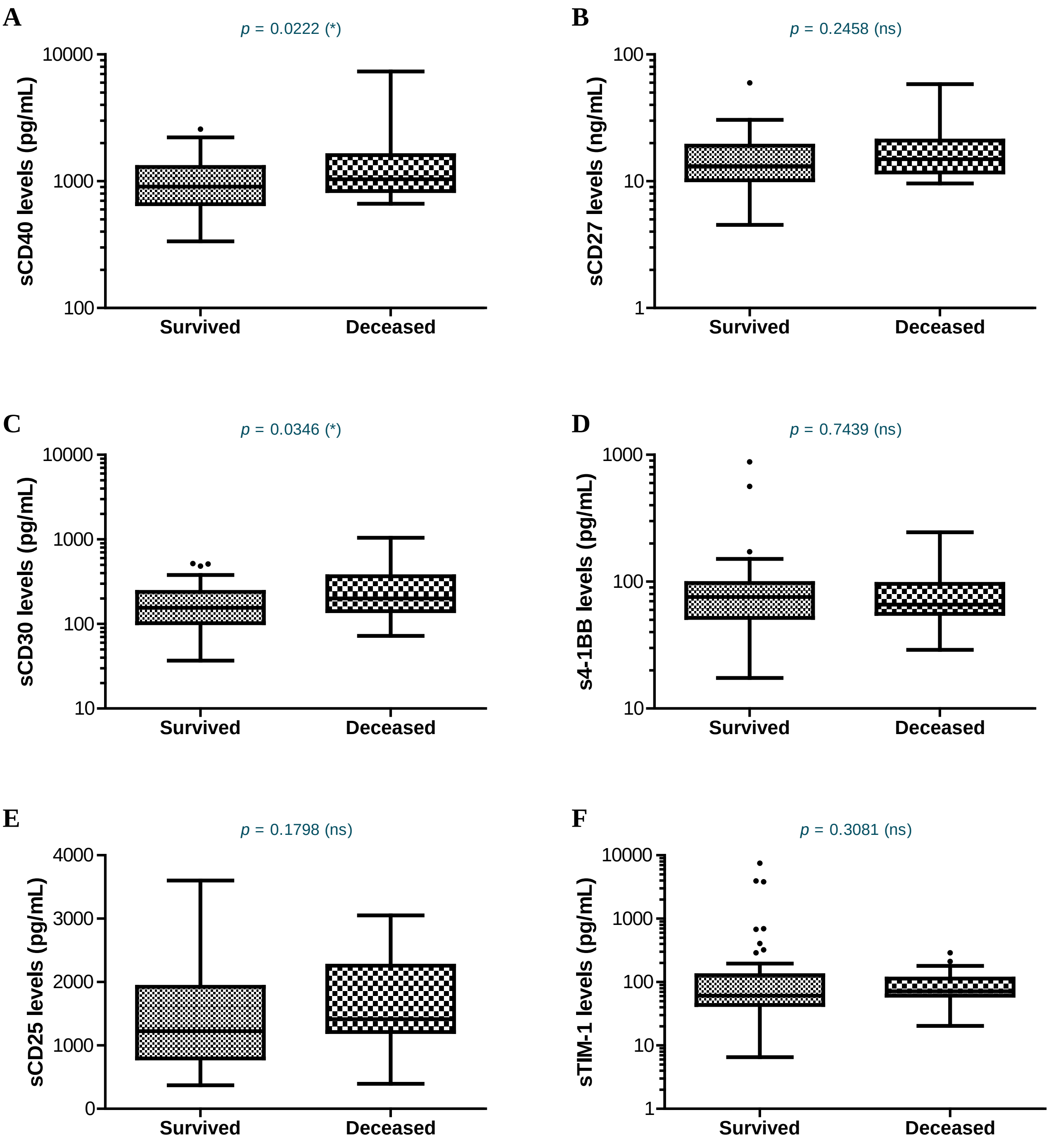

Supplementary Figure 3. Serum profile of soluble immune checkpoint receptors in severe COVID-19 stratified based on the survival outcome. Box and whiskers representation of **(A)** sCD40, **(B)** sCD27, **(C)** sCD30, **(D)** s4-1BB, **(E)** sCD25, **(F)** sTIM-1 serum levels for each category of severe COVID-19 disease: survived or deceased (*$p < 0.05$, ns – not significant; Kruskal-Wallis with Dunn's Multiple Comparison test).

Supplementary Figure 4

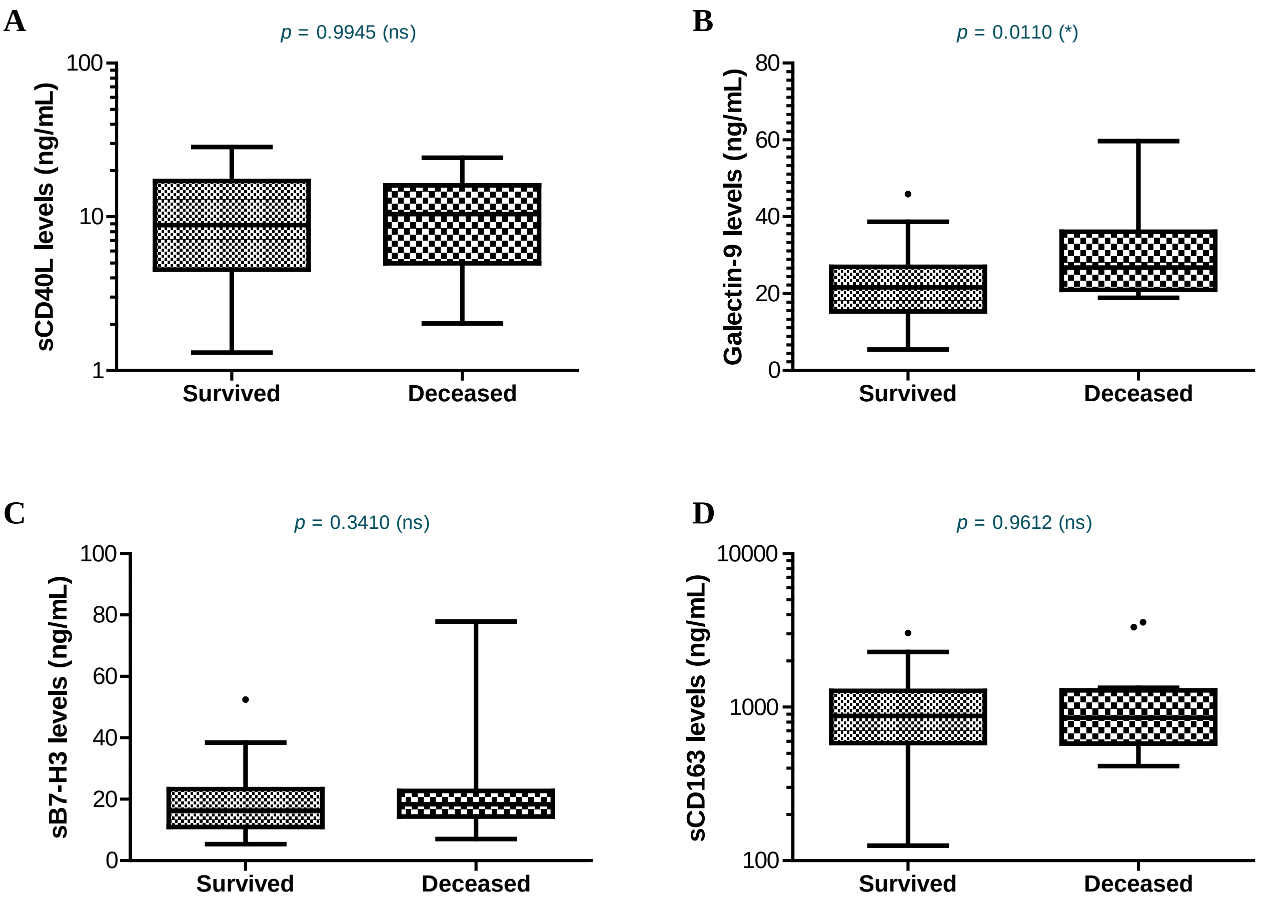

Supplementary Figure 4. Serum profile of soluble immune checkpoint ligands and sCD163 in severe COVID-19 stratified based on the survival outcome. Box and whiskers representation of **(A)** sCD40L, **(B)** Galectin 9, **(C)** sB7-H3, **(D)** sCD163 serum levels for each category of severe COVID-19 disease: survived or deceased (*$p < 0.05$, ns – not significant; Kruskal-Wallis with Dunn's Multiple Comparison test).

Supplementary Figure 5

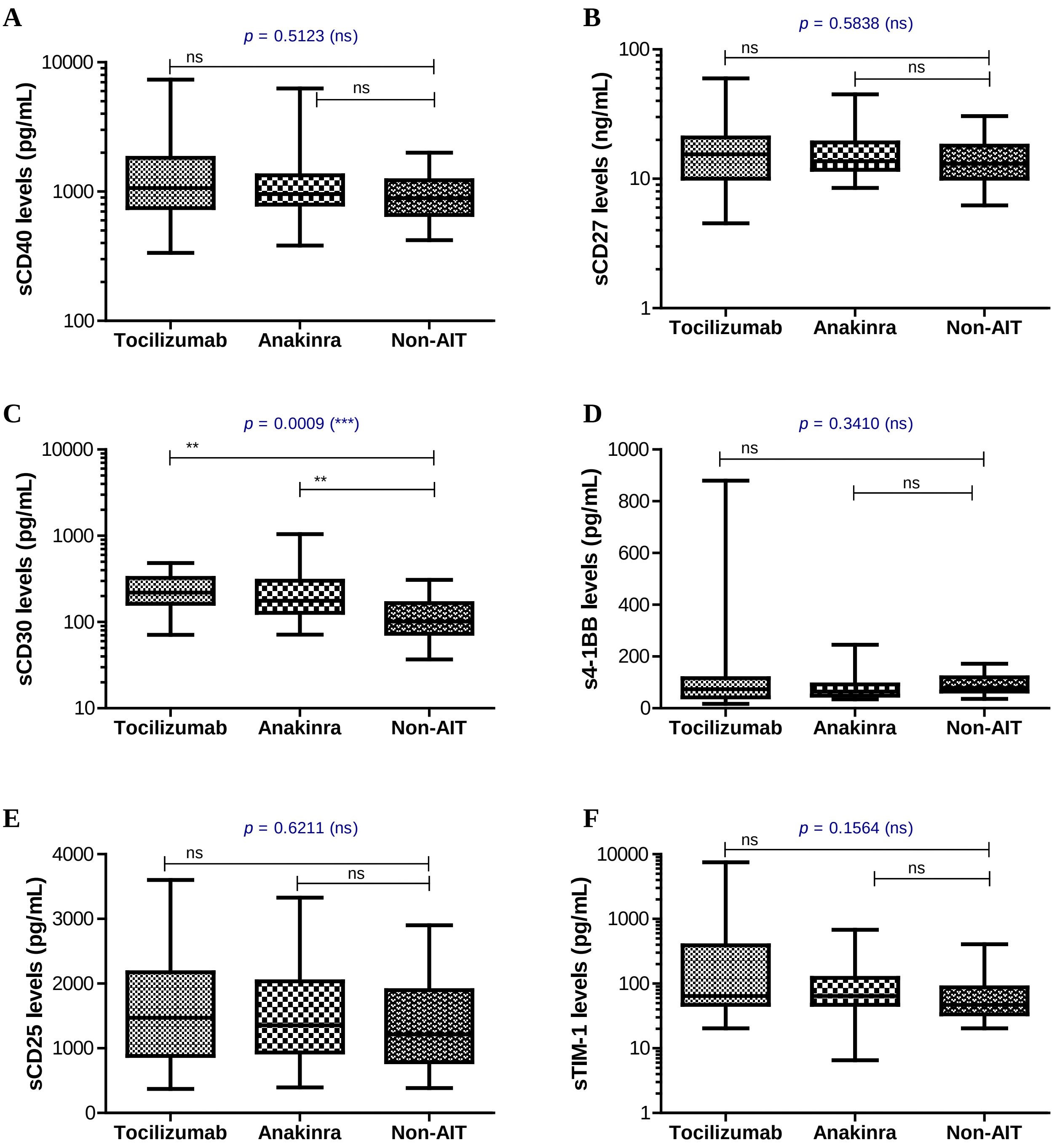

Supplementary Figure 5. Serum profile of soluble immune checkpoint receptors in severe COVID-19 cases receiving Tocilizumab, Anakinra or non-anti-interleukin medication. Box and whiskers representation of **(A)** sCD40, **(B)** sCD27, **(C)** **s**CD30, **(D)** **s**4-1BB, **(E)** sCD25, **(F)** sTIM-1 serum levels for each treatment category (***$p < 0.001$, **$p < 0.01$, ns – not significant; Kruskal-Wallis with Dunn's Multiple Comparison test).

Supplementary Figure 6

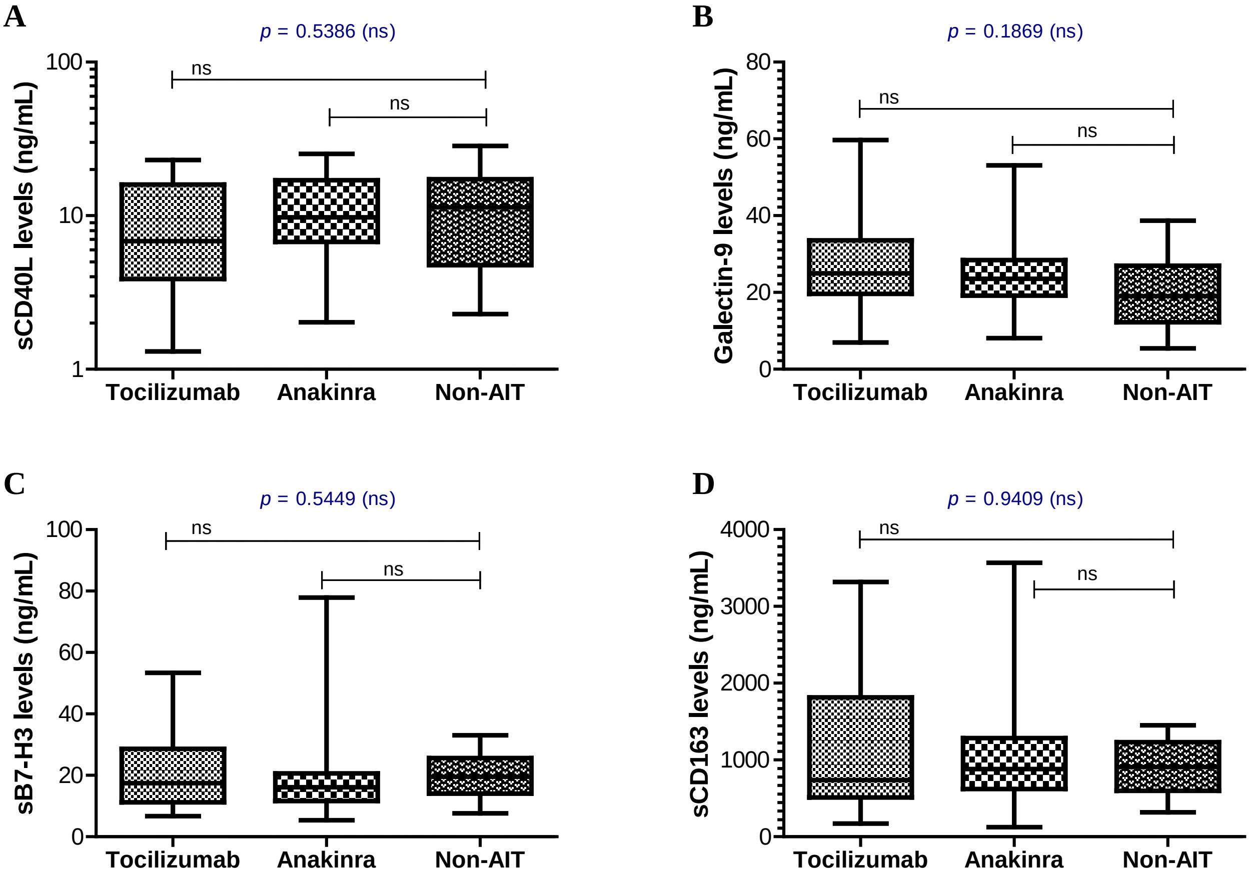

Supplementary Figure 6. Serum profile of soluble immune checkpoint ligands and sCD163 in severe COVID-19 cases receiving Tocilizumab, Anakinra or non-anti-interleukin medication. Box and whiskers representation of **(A)** sCD40L, **(B)** Galectin 9, **(C)** sB7-H3, **(D)** **s**CD163 serum levels for each treatment category (***$p < 0.001$, **$p < 0.01$, ns – not significant; Kruskal-Wallis with Dunn's Multiple Comparison test).

Supplementary Figure 7

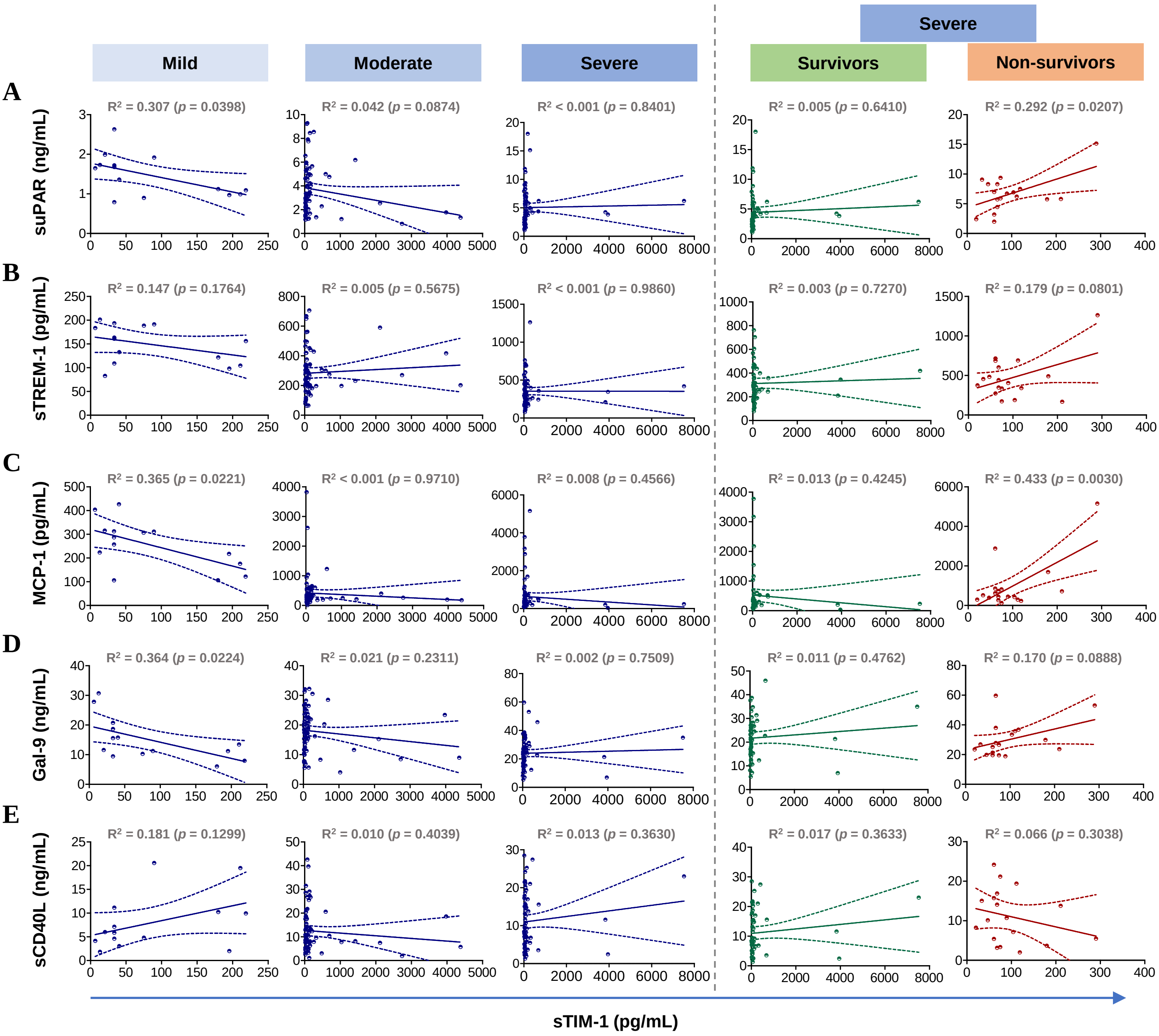

Supplementary Figure 7. Regression statistics describing the association between sTIM-1 and other soluble inflammatory biomarkers in mild, moderate and severe cases of SARS-CoV-2 infection. Linear regression analysis for **(A)** sTIM-1 and suPAR levels, **(B)** sTIM-1 and sTREM-1 levels, **(C)** sTIM-1 and MCP-1 levels, (**D**) sTIM-1 and Galectin-9 levels, and **(E)** sTIM-1 and sCD40L levels in mild, moderate and severe COVID-19 patients (Spearman test). The green lines and dots correspond to survivors, while the red lines and dots state for non-survivors.

Supplementary Figure 8

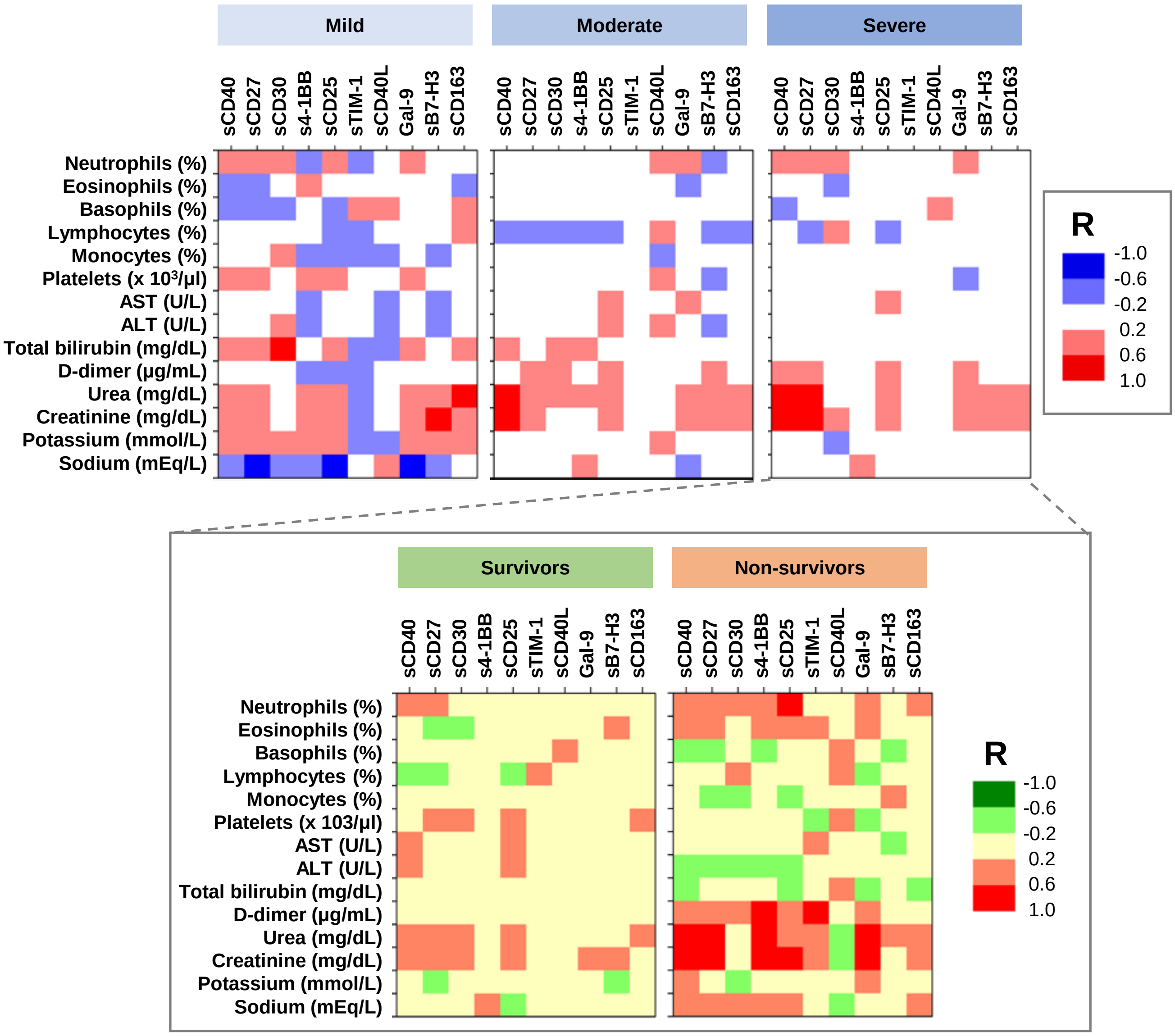

Supplementary Figure 8. Heat map describing the association between soluble immune checkpoint molecules and previously investigated paraclinical biomarkers in mild, moderate and severe cases of SARS-CoV-2 infection. The graph from the bottom depicts the differences in various associations between survivors and non-survivors (R = correlation coefficient).

Supplementary Figure 9

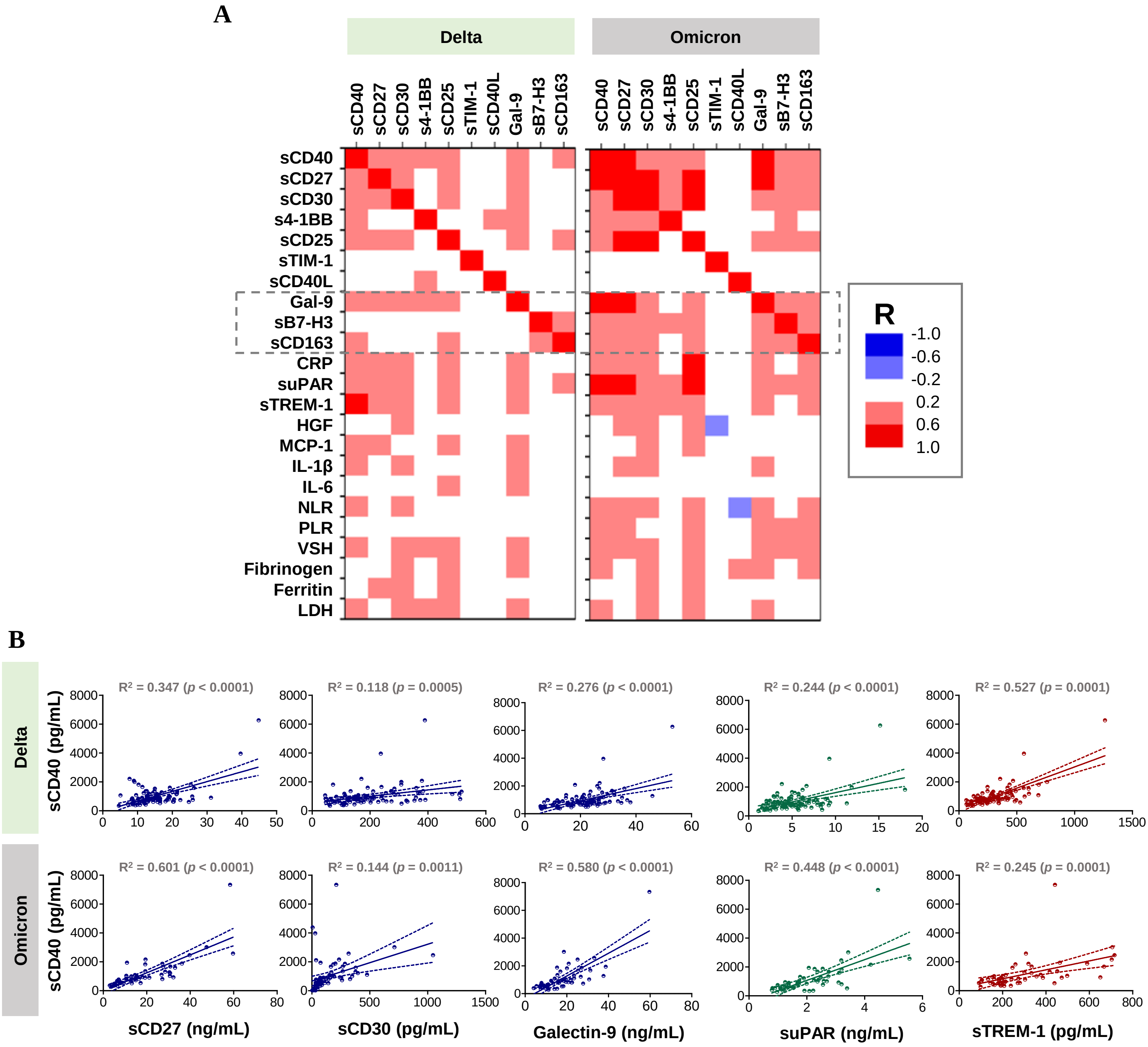

Supplementary Figure 9. Regression statistics describing the association between soluble immune checkpoint molecules and previously investigated paraclinical biomarkers in Delta and Omicron SARS-CoV-2 variant infections. (A) Heat map of correlation coefficients. **(B)** Linear regression analysis for CD40 and CD27/ CD30/ Galectin-9/ suPAR/ sTREM-1 in Delta (upper graphs) and Omicron (bottom graphs) infections (Spearman test).

Supplementary Figure 10

A

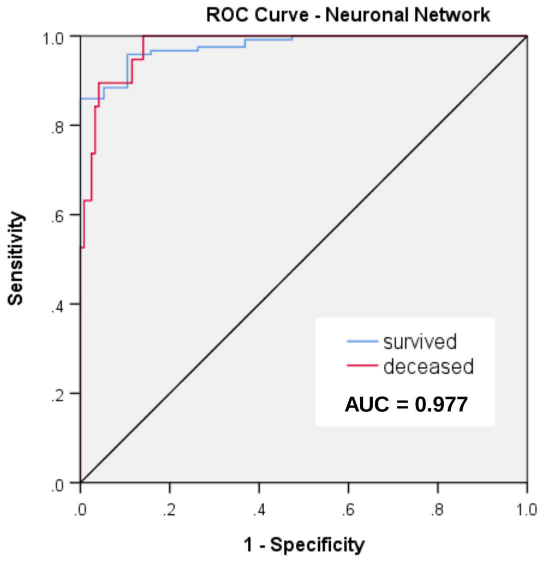

B

Parameters	Importance	Normalized Importance
**suPAR (ng/mL)**	0.108	100.0%
**sTREM-1 (pg/ml)**	0.087	80.4%
**sCD30 (pg/mL)**	0.075	69.7%
**Anti-interleukin therapy**	0.069	63.4%
**Age (years)**	0.063	58.4%
**Galectin 9 (ng/mL)**	0.063	58.2%
**sTIM 1 (pg/mL)**	0.062	57.4%
s4-1BB (pg/mL)	0.057	52.6%
Creatinine (mg/dL)	0.056	52.2%
sB7-H3 (ng/mL)	0.056	51.3%
sCD163 (ng/mL)	0.046	42.6%
sCD27 (ng/mL)	0.046	42.4%
ICD10 - K	0.037	34.3%
sCD25 (pg/mL)	0.035	32.0%
sCD40 (pg/mL)	0.030	27.4%
Urea (mg/dL)	0.029	26.8%
ICD10 - D	0.019	17.3%
sCD40L (ng/mL)	0.014	13.0%
ICD10 - E	0.013	12.0%
ICD10 - I	0.011	10.3%
Vaccination (no)	0.009	8.3%
MCP-1 (pg/ml)	0.008	7.3%
Male	0.007	6.3%

Supplementary Figure 10. Neural network models of outcome prediction. (A) ROC analysis of the neural network model based on the serum levels of soluble immune checkpoint molecules and the previously reported predictors of mortality (suPAR, sTREM-1, MCP-1), corrected for the therapy supplied during hospitalization, the presence of comorbidities, vaccination status, age and gender. **(B)** The importance of each variable in the generated neural network model.

Supplementary Figure 11

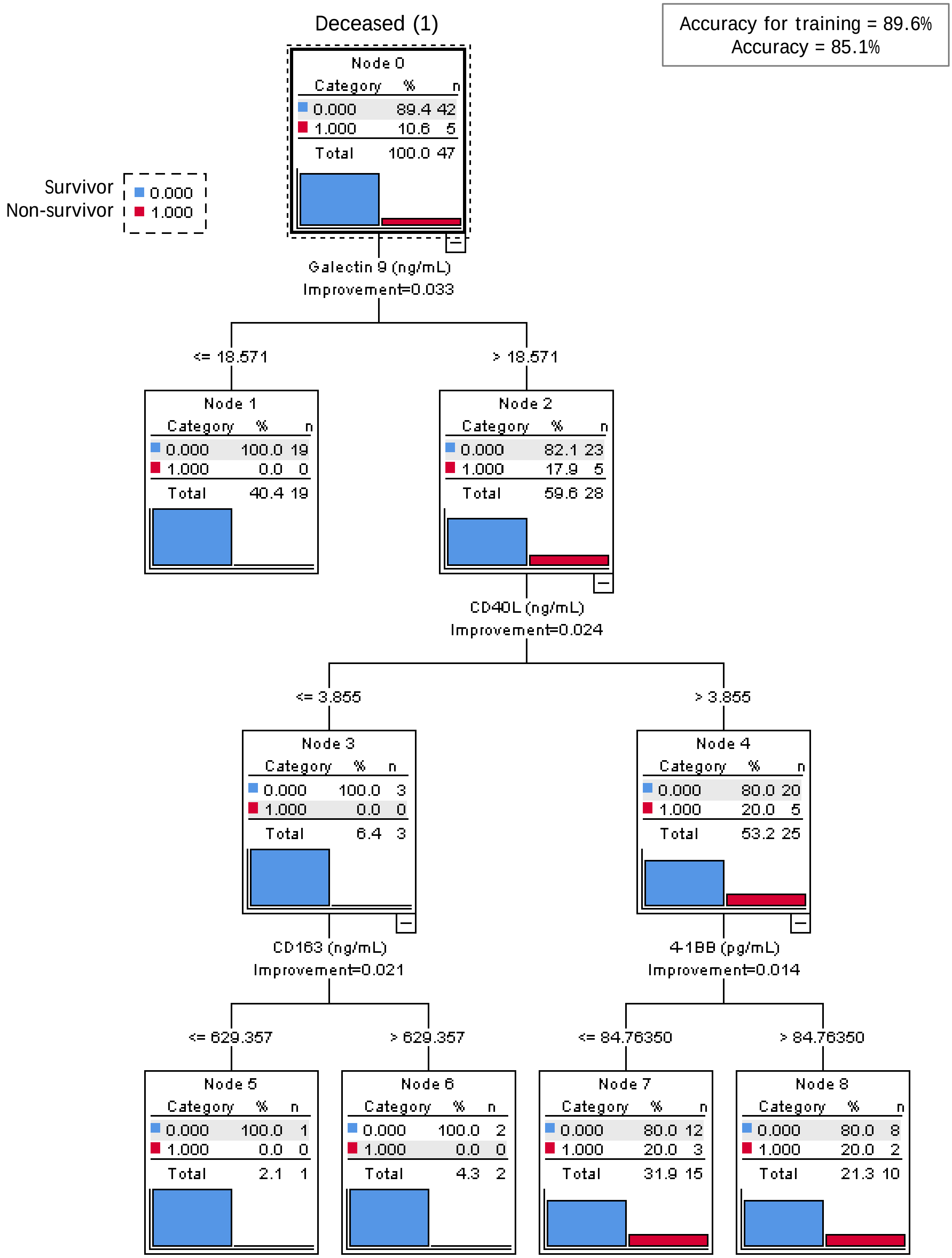

Supplementary Figure 11. Decision tree analysis of soluble immune checkpoint molecules. Survivors are denoted as 0, while non-survivors/deceased as 1.

Supplementary Figure 12

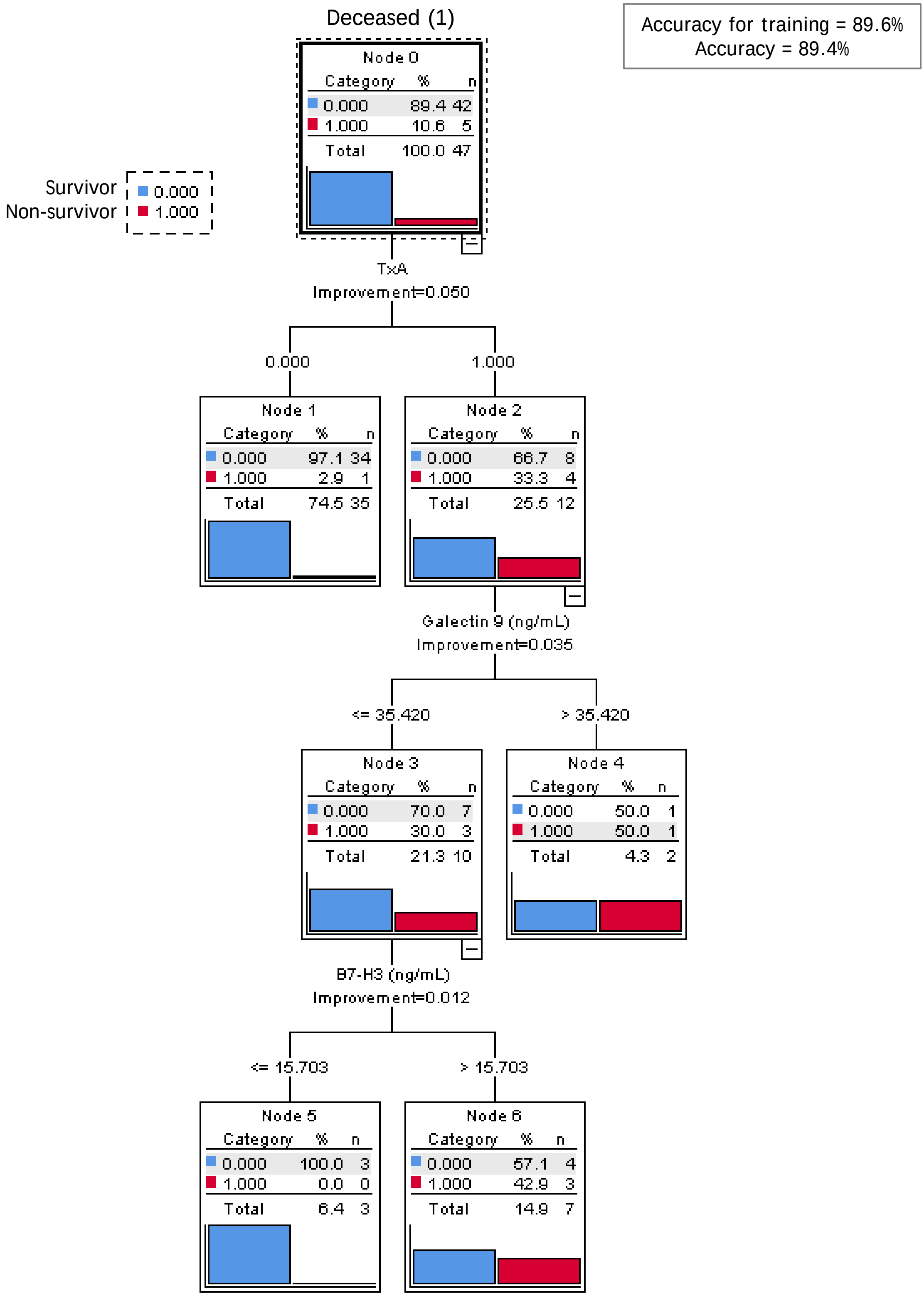

Supplementary Figure 12. Decision tree analysis of soluble immune checkpoint molecules when corrected for confounders. The clinical/paraclinical confounders included: anti-interleukin therapy with Tocilizumab or Anakinra (TxA, 0 = no, 1 = yes), age, gender, vaccination status, comorbidities. Survivors are denoted as 0, while non-survivors/deceased as 1.

Supplementary Table 1. Patients' characteristics

Parameters	Mild		Moderate		Severe		*p*-value
	Delta	Omicron	Delta	Omicron	Delta	Omicron	
**Cases (n, %)**<br>**M (n, %)**<br>**F (n, %)**	3 (1.96%)<br>1 (33.3%)<br>2 (66.6%)	11 (7.19%)<br>4 (36.4%)<br>7 (63.6%)	46 (30.1%)<br>25 (54.3%)<br>21 (45.7%)	25 (16.3%)<br>15 (60%)<br>10 (40%)	49 (32%)<br>29 (59.2%)<br>20 (40.8%)	19 (12.41%)<br>14 (73.7%)<br>5 (26.3%)	0.4071
**Age (median, IQR)**	51 [45-59]	56 [46-71]	67 [56-72]	68 [62.5-77.5]	70 [59-78]	69 [52-79]	0.1786
**Vaccinated (n, %)**	2 (66.6%)	7 (63.6%)	7 (15.2%)	9 (36%)	6 (12.2%)	6 (31.6%)	**0.0016**
**Total days of disease**	11 [10-15]	11 [9-15]	17 [15-20.25]	15 [10-19.5]	21 [16-29.5]	17 [8-23]	**< 0.0001**
**Days of symptoms before hospitalization (median, IQR)**	5 [1-10]	4 [2-6]	7 [5-10]	4 [1-6.5]	7 [5-10]	6 [2-8]	**0.0093**
**Hospitalization days (median, IQR)**	6 [5-9]	6 [5-10]	10 [7.75-11]	10 [6-13.5]	14 [8.5-20]	10 [5-15]	**< 0.0001**
**Death rate (n, %)**	0 (0%)	0 (0%)	1 (2.17%)	1 (4%)	14(28.6%)	4 (21.1%)	**0.0012**

Abbreviations: n, number of cases; IQR, interquartile range; *p*, statistical significance coefficient.

Supplementary Table 2. Statistical evaluation of soluble IC molecules for predicting COVID-19 severity.

Variable	AUC	S.E.M.	*p*-value	95% CI
**sCD40 (pg/mL)**	0.666	0.052	**0.003**	0.563-0.768
sCD27 (ng/mL)	0.579	0.056	0.160	0.469-0.688
sCD30 (pg/mL)	0.587	0.056	0.122	0.477-0.697
s4-1BB (pg/mL)	0.567	0.057	0.232	0.456-0.678
**sCD25 (pg/mL)**	0.682	0.052	**0.001**	0.580-0.782
sTIM-1 (pg/mL)	0.538	0.058	0.501	0.425-0.650
sCD40L (ng/mL)	0.537	0.057	0.513	0.425-0.648
**Galectin-9 (ng/mL)**	0.718	0.049	**0.000**	0.621-0.814
sB7-H3 (ng/mL)	0.530	0.056	0.591	0.419-0.640
sCD163 (ng/mL)	0.562	0.056	0.273	0.451-0.672

Abbreviations: sTIM-1, soluble T cell immunoglobulin domain and the mucin domain protein-1; sCD40L, soluble CD40 ligand; AUC, area under curve; SEM, standard error of the mean; CI, confidence interval; *p*, statistical significance coefficient.

Supplementary Table 3. Statistical evaluation of clinical and paraclinical parameters in association with severe COVID-19.

Variable	AUC	S.E.M.	*p*-value	95% CI
Laboratory analyte				
**CRP (mg/L)**	0.737	0.050	**0.000**	0.640-0.834
**ESR (mm/h)**	0.661	0.055	**0.005**	0.554-0.768
**Fibrinogen (g/L)**	0.713	0.051	**0.000**	0.612-0.812
**Ferritin (µg/L)**	0.645	0.055	**0.013**	0.536-0.752
**LDH (U/L)**	0.719	0.051	**0.000**	0.618-0.818
**Neutrophils (x 10^3/µl)**	0.645	0.056	**0.013**	0.535-0.753
**Neutrophils (%)**	0.736	0.050	**0.000**	0.637-0.834
**Eosinophils (x 10^3/µl)**	0.376	0.057	**0.033**	0.264-0.486
**Eosinophils (%)**	0.369	0.056	**0.024**	0.258-0.479
Basophils (x 10^3/µl)	0.441	0.058	0.313	0.328-0.554
Basophils (%)	0.402	0.057	0.092	0.290-0.513
**Lymphocytes (x 10^3/µl)**	0.326	0.057	**0.003**	0.214-0.436
**Lymphocytes (%)**	0.295	0.053	**0.000**	0.191-0.399
Monocytes (x 10^3/µl)	0.434	0.058	0.257	0.320-0.547
**Monocytes (%)**	0.324	0.054	**0.002**	0.218-0.429
Hemoglobin (g/dL)	0.553	0.058	0.366	0.439-0.665
Hematocrit (%)	0.541	0.058	0.485	0.426-0.654
Platelets (x 10^3/µl)	0.558	0.058	0.318	0.444-0.671
Total serum protein (g/L)	0.498	0.059	0.967	0.381-0.613
AST (U/L)	0.601	0.058	0.082	0.488-0.713
ALT (U/L)	0.520	0.059	0.725	0.404-0.636
Total bilirubin (mg/dL)	0.450	0.058	0.394	0.335-0.565
Prothrombin index	0.455	0.058	0.439	0.340-0.569
**D-dimer (µg/mL)**	0.696	0.053	**0.001**	0.592-0.799
**Blood glucose (mg/dL)**	0.615	0.057	**0.048**	0.503-0.726
**Urea (mg/dL)**	0.680	0.054	**0.002**	0.574-0.785
Creatinine (mg/dL)	0.612	0.057	0.054	0.500-0.723
**Potassium (mmol/L)**	0.660	0.055	**0.006**	0.552-0.766
**Sodium (mmol/L)**	0.346	0.055	**0.008**	0.238-0.453
Ionized calcium (mg/dL)	0.513	0.058	0.825	0.398-0.626
Chloride (mmol/L)	0.429	0.058	0.222	0.315-0.542
Patients' characteristics				
Delta variant infection	0.621	0.057	**0.038**	0.509-0.731
Age (years)	0.626	0.056	**0.030**	0.516-0.736
Gender (M)	0.502	0.058	0.978	0.387-0.615
Vaccination status (no)	0.543	0.058	0.462	0.428-0.656
Days of symptoms prior hospitalization	0.588	0.057	0.132	0.475-0.699
Comorbidities (ICD 10)				
ICD10 – D	0.494	0.058	0.912	0.379-0.607
ICD10 – E	0.543	0.058	0.458	0.430-0.656
ICD10 – I	0.552	0.058	0.371	0.438-0.665
ICD10 – K	0.516	0.058	0.777	0.402-0.630

Abbreviations: CRP, C-reactive protein; ESR, erythrocyte sedimentation rate; LDH, lactate dehydrogenase; WBC, white blood cells; AST, aspartate transaminase; ALT, alanine transaminase; ICD10 – D, diseases of the blood involving the immune mechanism (anemia, purpura and other hemorrhagic conditions); ICD10 – E, endocrine and metabolic disorders, ICD10 – I, diseases of the circulatory system, ICD10 – K, diseases of liver and gallbladder; AUC, area under curve; CI, confidence interval; *p*, statistical significance coefficient.

Supplementary Table 4. Multivariate regression analysis of soluble IC molecules as predictors for severe COVID-19 in relation to paraclinical and clinical confounders.

Variable	HR	95% CI	*p*-value
Laboratory analyte			
sCD40 (pg/mL)	1.000	0.998-1.001	0.593
sCD25 (pg/mL)	1.000	0.999-1.001	0.466
Galectin-9 (ng/mL)	1.035	0.942-1.136	0.471
CRP (mg/L)	0.999	0.984-1.012	0.865
ESR (mm/h)	0.982	0.958-1.006	0.157
Fibrinogen (g/L)	1.962	0.972-3.957	0.060
Ferritin (μg/L)	1.000	0.999-1.000	0.231
LDH (U/L)	1.005	0.998-1.010	0.150
Neutrophils (%)	2.910	0.231-36.63	0.409
Eosinophils (%)	3.861	0.236-62.92	0.343
Lymphocytes (%)	3.002	0.233-38.51	0.399
Monocytes (%)	2.484	0.188-32.80	0.490
D-dimer (μg/mL)	1.314	0.739-2.333	0.351
Blood glucose (mg/dL)	1.003	0.995-1.011	0.405
Urea (mg/dL)	0.998	0.974-1.023	0.897
Potassium (mmol/L)	2.406	0.810-7.145	0.114
Sodium (mmol/L)	0.911	0.737-1.124	0.383
Patients' characteristics			
Delta variant infection	1.743	0.494-6.140	0.387
Age (years)	1.035	0.990-1.081	0.122

Abbreviations: CRP, C-reactive protein; ESR, erythrocyte sedimentation rate; LDH, lactate dehydrogenase; HR, hazard ratio; CI, confidence interval; *p*, statistical significance coefficient.

Supplementary Table 5. Statistical evaluation of soluble IC molecules in association with Delta variant infection.

Variable	AUC	S.E.M.	*p*-value	95% CI
sCD40 (pg/mL)	0.496	0.052	0.927	0.392-0.598
sCD27 (ng/mL)	0.478	0.056	0.651	0.368-0.587
**sCD30 (pg/mL)**	0.600	0.051	**0.040**	0.499-0.700
s4-1BB (pg/mL)	0.551	0.052	0.299	0.449-0.651
sCD25 (pg/mL)	0.533	0.053	0.499	0.429-0.636
**sTIM-1 (pg/mL)**	0.610	0.049	**0.024**	0.514-0.706
**sCD40L (ng/mL)**	0.685	0.044	**0.000**	0.598-0.771
**Galectin-9 (ng/mL)**	0.629	0.048	**0.008**	0.535-0.722
**sB7-H3 (ng/mL)**	0.308	0.045	**0.000**	0.220-0.394
**sCD163 (ng/mL)**	0.403	0.049	**0.048**	0.307-0.499

Abbreviations: sTIM-1, soluble T cell immunoglobulin domain and the mucin domain protein-1; sCD40L, soluble CD40 ligand; AUC, area under curve; SEM, standard error of the mean; CI, confidence interval; *p*, statistical significance coefficient.

Supplementary Table 6. Statistical evaluation of clinical and paraclinical parameters in association with Delta variant infection.

Variable	AUC	S.E.M.	*p*-value	95% CI
Laboratory analyte				
CRP (mg/L)	0.575	0.064	0.219	0.450-0.699
**ESR (mm/h)**	0.627	0.061	**0.037**	0.507-0.747
**Fibrinogen (g/L)**	0.640	0.064	**0.022**	0.514-0.765
Ferritin (µg/L)	0.541	0.064	0.503	0.415-0.666
**LDH (U/L)**	0.674	0.061	**0.004**	0.553-0.794
Neutrophils (x 10^3/µl)	0.590	0.061	0.144	0.470-0.708
Neutrophils (%)	0.585	0.063	0.163	0.462-0.708
Eosinophils (x 10^3/µl)	0.401	0.061	0.107	0.282-0.520
Eosinophils (%)	0.395	0.061	0.088	0.276-0.514
Basophils (x 10^3/µl)	0.504	0.061	0.945	0.384-0.624
Basophils (%)	0.496	0.061	0.942	0.375-0.615
Lymphocytes (x 10^3/µl)	0.464	0.063	0.560	0.340-0.588
Lymphocytes (%)	0.441	0.063	0.331	0.317-0.563
Monocytes (x 10^3/µl)	0.515	0.061	0.810	0.394-0.634
Monocytes (%)	0.456	0.065	0.471	0.327-0.584
Hemoglobin (g/dL)	0.575	0.066	0.223	0.445-0.704
Hematocrit (%)	0.563	0.068	0.307	0.429-0.695
Platelets (x 10^3/µl)	0.606	0.059	0.084	0.489-0.722
Total serum protein (g/L)	0.546	0.063	0.449	0.422-0.670
AST (U/L)	0.610	0.062	0.073	0.488-0.730
ALT (U/L)	0.516	0.064	0.799	0.390-0.641
Total bilirubin (mg/dL)	0.586	0.063	0.161	0.461-0.710
Prothrombin index	0.551	0.063	0.405	0.428-0.673
D-dimer (µg/mL)	0.546	0.066	0.451	0.417-0.675
Blood glucose (mg/dL)	0.415	0.061	0.165	0.295-0.534
Urea (mg/dL)	0.462	0.064	0.539	0.337-0.587
Creatinine (mg/dL)	0.431	0.063	0.261	0.307-0.554
**Potassium (mmol/L)**	0.642	0.058	**0.020**	0.529-0.755
Sodium (mmol/L)	0.407	0.057	0.130	0.294-0.519
Ionized calcium (mg/dL)	0.394	0.058	0.084	0.279-0.508
Chloride (mmol/L)	0.489	0.061	0.861	0.369-0.608
Patients' characteristics				
Age (years)	0.498	0.049	0.974	0.401-0.595
Gender (M)	0.481	0.049	0.691	0.385-0.576
**Vaccination status (no)**	0.623	0.049	**0.011**	0.527-0.719
**Days of symptoms prior hospitalization**	0.686	0.046	**0.000**	0.596-0.776
Comorbidities (ICD 10)				
**ICD10 – D**	0.399	0.049	**0.038**	0.302-0.495
ICD10 – E	0.488	0.049	0.806	0.391-0.584
ICD10 – I	0.474	0.048	0.598	0.379-0.569
ICD10 – K	0.467	0.049	0.499	0.370-0.563

Abbreviations: CRP, C-reactive protein; ESR, erythrocyte sedimentation rate; LDH, lactate dehydrogenase; WBC, white blood cells; AST, aspartate transaminase; ALT, alanine transaminase; ICD10 – D, diseases of the blood involving the immune mechanism (anemia, purpura and other hemorrhagic conditions); ICD10 – E, endocrine and metabolic disorders, ICD10 – I, diseases of the circulatory system, ICD10 – K, diseases of liver and gallbladder; AUC, area under curve; CI, confidence interval; *p*, statistical significance coefficient.

Supplementary Table 7. Multivariate regression analysis of soluble IC molecules as predictors for Delta variant infection in relation to paraclinical and clinical confounders.

Variable	HR	95% CI	*p*-value
Laboratory analyte			
sCD30 (pg/mL)	0.999	0.993-1.004	0.704
sTIM-1 (pg/mL)	1.000	0.999-1.000	0.096
sCD40L (ng/mL)	1.088	0.986-1.200	0.091
Galectin-9 (ng/mL)	1.118	1.036-1.206	**0.004**
sB7-H3 (ng/mL)	0.963	0.918-1.010	0.123
sCD163 (ng/mL)	0.999	0.997-0.999	**0.023**
ESR (mm/h)	0.995	0.971-1.019	0.693
Fibrinogen (g/L)	1.189	0.666-2.120	0.557
LDH (U/L)	0.999	0.996-1.001	0.575
Potassium (mmol/L)	3.609	1.188-10.95	**0.024**
Patients' characteristics			
Vaccination status (no)	3.884	1.107-13.62	**0.034**
Days of symptoms prior hospitalization	1.099	0.932-1.295	0.258
Comorbidities (ICD10 – D)	0.223	0.069-0.714	**0.012**

Abbreviations: ESR, erythrocyte sedimentation rate; LDH, lactate dehydrogenase; ICD10 – D, diseases of the blood involving the immune mechanism (anemia, purpura and other hemorrhagic conditions); HR, hazard ratio; CI, confidence interval; *p*, statistical significance coefficient.

Supplementary Table 8. Statistical evaluation of soluble IC molecules for predicting COVID-19 mortality.

Variable	AUC	S.E.M.	*p*-value	95% CI	Cut-off	Sensitivity	Specificity
**sCD40 (pg/mL)**	0.709	0.054	**0.003**	0.602-0.814	931.12	0.650	0.654
sCD27 (ng/mL)	0.625	0.063	0.071	0.501-0.748			
**sCD30 (pg/mL)**	0.664	0.062	**0.019**	0.542-0.784	181.98	0.600	0.609
s4-1BB (pg/mL)	0.541	0.068	0.559	0.408-0.673			
**sCD25 (pg/mL)**	0.695	0.059	**0.005**	0.580-0.810	1164.39	0.850	0.564
sTIM-1 (pg/mL)	0.564	0.053	0.358	0.459-0.668			
sCD40L (ng/mL)	0.493	0.072	0.920	0.352-0.634			
**Galectin-9 (ng/mL)**	0.773	0.047	**0.000**	0.681-0.865	23.46	0.700	0.707
sB7-H3 (ng/mL)	0.568	0.067	0.329	0.436-0.699			
sCD163 (ng/mL)	0.514	0.068	0.837	0.381-0.647			

Abbreviations: sTIM-1, soluble T cell immunoglobulin domain and the mucin domain protein-1, sCD40L, soluble CD40 ligand; AUC, area under curve; SEM, standard error of the mean; CI, confidence interval; *p*, statistical significance coefficient.

Supplementary Table 9. Statistical evaluation of clinical and paraclinical parameters in association with COVID-19 mortality.

Variable	AUC	S.E.M.	*p*-value	95% CI
Laboratory analyte				
**CRP (mg/L)**	0.702	0.077	**0.013**	0.550-0.853
ESR (mm/h)	0.618	0.069	0.148	0.481-0.753
Fibrinogen (g/L)	0.619	0.066	0.144	0.489-0.748
**Ferritin (μg/L)**	0.723	0.057	**0.006**	0.611-0.835
**LDH (U/L)**	0.807	0.055	**0.000**	0.699-0.914
Neutrophils (x 10^3/μl)	0.543	0.095	0.595	0.357-0.728
**Neutrophils (%)**	0.673	0.075	**0.033**	0.526-0.820
Eosinophils (x 10^3/μl)	0.365	0.065	0.096	0.236-0.492
Eosinophils (%)	0.361	0.064	0.087	0.234-0.486
Basophils (x 10^3/μl)	0.438	0.087	0.446	0.268-0.607
Basophils (%)	0.431	0.085	0.393	0.263-0.597
**Lymphocytes (x 10^3/μl)**	0.254	0.063	**0.002**	0.131-0.376
**Lymphocytes (%)**	0.329	0.072	**0.035**	0.187-0.469
Monocytes (x 10^3/μl)	0.402	0.077	0.228	0.251-0.552
Monocytes (%)	0.421	0.093	0.330	0.238-0.602
Hemoglobin (g/dL)	0.617	0.092	0.149	0.437-0.797
Hematocrit (%)	0.600	0.087	0.217	0.429-0.771
**Platelets (x 10^3/μl)**	0.328	0.070	**0.034**	0.191-0.464
Total serum protein (g/L)	0.473	0.073	0.739	0.329-0.616
**AST (U/L)**	0.736	0.060	**0.004**	0.618-0.852
ALT (U/L)	0.599	0.059	0.222	0.483-0.715
Total bilirubin (mg/dL)	0.436	0.089	0.434	0.261-0.611
Prothrombin index	0.601	0.072	0.213	0.459-0.743
**D-dimer (μg/mL)**	0.709	0.078	**0.010**	0.556-0.860
Blood glucose (mg/dL)	0.634	0.073	0.099	0.491-0.776
**Urea (mg/dL)**	0.720	0.068	**0.007**	0.585-0.853
Creatinine (mg/dL)	0.641	0.085	0.082	0.474-0.807
Potassium (mmol/L)	0.626	0.093	0.121	0.443-0.808
Sodium (mmol/L)	0.346	0.076	0.058	0.196-0.495
Ionized calcium (mg/dL)	0.353	0.078	0.071	0.199-0.506
Chloride (mmol/L)	0.453	0.095	0.566	0.267-0.639
Patients' characteristics				
Delta variant infection	0.563	0.066	0.365	0.433-0.693
Age (years)	0.617	0.060	0.092	0.498-0.735
Gender (M)	0.543	0.068	0.535	0.409-0.676
Vaccination status (no)	0.495	0.070	0.946	0.358-0.631
Days of symptoms prior hospitalization	0.587	0.062	0.212	0.465-0.708
Comorbidities (ICD 10)				
ICD10 – D	0.604	0.074	0.135	0.459-0.747
ICD10 – E	0.496	0.070	0.959	0.359-0.633
ICD10 – I	0.571	0.068	0.305	0.438-0.703
ICD10 – K	0.551	0.074	0.460	0.406-0.695

Abbreviations: CRP, C-reactive protein; ESR, erythrocyte sedimentation rate; LDH, lactate dehydrogenase; WBC, white blood cells; AST, aspartate transaminase; ALT, alanine transaminase; ICD10 – D, diseases of the blood involving the immune mechanism (anemia, purpura and other hemorrhagic conditions); ICD10 – E, endocrine and metabolic disorders, ICD10 – I, diseases of the circulatory system, ICD10 – K, diseases of liver and gallbladder; AUC, area under curve; CI, confidence interval; *p*, statistical significance coefficient.

Supplementary Table 10. Multivariate regression analysis of soluble IC molecules as prognostic factors for COVID-19 in relation to paraclinical confounders.

Variable	HR	95% CI	*p*-value
Laboratory analyte			
sCD40 (pg/mL)	1.000	0.998-1.002	0.574
sCD30 (pg/mL)	1.001	0.995-1.005	0.825
sCD25 (pg/mL)	1.000	0.998-1.001	0.827
Galectin-9 (ng/mL)	1.046	0.949-1.152	0.362
CRP (mg/L)	1.004	0.990-1.017	0.590
Ferritin (µg/L)	0.999	0.998-1.000	0.156
LDH (U/L)	1.005	1.000-1.010	**0.046**
Neutrophils (%)	0.886	0.734-1.069	0.207
Lymphocytes (%)	0.804	0.619-1.043	0.101
Platelets (x 10^3/µl)	0.993	0.983-1.001	0.092
AST (U/L)	1.001	0.987-1.014	0.885
D-dimer (µg/mL)	1.177	0.693-1.996	0.547
Urea (mg/dL)	1.003	0.973-1.033	0.839

Abbreviations: CRP, C-reactive protein; LDH, lactate dehydrogenase; AST, aspartate transaminase; HR, hazard ratio; CI, confidence interval; *p*, statistical significance coefficient.

Supplementary Table 11. Cellular expression of immune checkpoint molecules.

	Activated CD4+ T cells	Activated CD8+ T cells	Tregs	NK cells	B cells	Dendritic cells	Macrophages	Monocytes	Other cells
**CD40**									endothelial and epithelial cells
**CD27**									
**CD30**									
**4-1BB/ CD137**									
**CD25**									myeloid-derived suppressor cells
**TIM-1**									
**CD40L**									platelets, smooth muscle cells
**Galectin-9**									
**B7-H3/ CD276**									
**CD163**									

Legend: Co-stimulatory Co-inhibitory

Receptor Ligand
